# Tetherin Can Restrict Cell-Free and Cell-Cell Transmission of HIV from Primary Macrophages to T Cells

**DOI:** 10.1371/journal.ppat.1004189

**Published:** 2014-07-03

**Authors:** Sebastian Giese, Mark Marsh

**Affiliations:** MRC/UCL Laboratory for Molecular Cell Biology, University College London, London, United Kingdom; Vanderbilt University School of Medicine, United States of America

## Abstract

Bst-2/Tetherin inhibits the release of HIV by tethering newly formed virus particles to the plasma membrane of infected cells. Although the mechanisms of Tetherin-mediated restriction are increasingly well understood, the biological relevance of this restriction in the natural target cells of HIV is unclear. Moreover, whether Tetherin exerts any restriction on the direct cell-cell spread of HIV across intercellular contacts remains controversial. Here we analyse the restriction endogenous Tetherin imposes on HIV transmission from primary human macrophages, one of the main targets of HIV *in vivo*. We find that the mRNA and protein levels of Tetherin in macrophages are comparable to those in T cells from the same donors, and are highly upregulated by type I interferons. Improved immunocytochemistry protocols enable us to demonstrate that Tetherin localises to the cell surface, the *trans*-Golgi network, and the macrophage HIV assembly compartments. Tetherin retains budded virions in the assembly compartments, thereby impeding the release and cell-free spread of HIV, but it is not required for the maintenance of these compartments *per se*. Notably, using a novel assay to quantify cell-cell spread, we show that Tetherin promotes the transfer of virus clusters from macrophages to T cells and thereby restricts the direct transmission of a dual-tropic HIV-1. Kinetic analyses provide support for the notion that this direct macrophage-T cell spread is mediated, at least in part, by so-called virological synapses. Finally, we demonstrate that the viral Vpu protein efficiently downregulates the cell surface and overall levels of Tetherin, and thereby abrogates this HIV restriction in macrophages. Together, our study shows that Tetherin, one of the most potent HIV restriction factors identified to date, can inhibit virus spread from primary macrophages, regardless of the mode of transmission.

## Author Summary

Tetherin is a cellular protein that inhibits (or restricts) a broad range of enveloped viruses, including HIV, by physically “tethering” nascent particles to the plasma membrane of infected cells. CD4^+^ T cells and macrophages are the main targets of HIV *in vivo*, and both cell types express Tetherin. Although the mechanisms of Tetherin-mediated restriction in model cell lines and T cells are increasingly well understood, experimental data from macrophages are sparse, and partially contradict observations made in other cell types. Here we investigate the sensitivity of Tetherin expression to interferon, and the subcellular localisation of the restriction factor in primary human macrophages. We find that Tetherin inhibits HIV release by retaining nascent particles in macrophage HIV assembly compartments, and can also restrict the transmission of HIV across intercellular contacts between macrophages and T cells. Finally, we demonstrate that the HIV protein Vpu efficiently counteracts Tetherin in macrophages, and thereby ensures viral propagation. Our results, together with other published data, show that Tetherin can efficiently inhibit viral replication in both major target cell types of HIV, regardless of the mode of transmission. These data support the view that efficient counteraction of Tetherin was a crucial factor for the global spread of HIV.

## Introduction

The replication of viruses can be inhibited by a number of cellular proteins, collectively referred to as restriction factors [1]. In many cases, the expression of restriction factors is induced or enhanced by type I interferons (IFN), which are upregulated following infection with intracellular pathogens such as viruses. The primate lentiviruses, including human immunodeficiency viruses (HIV), are subject to restriction at multiple stages of their life cycles [1]. In a number of these cases, viruses have evolved mechanisms to abrogate the influence of specific cellular restriction factors. Recently, HM1.24/CD317/Bst-2/Tetherin (Ensembl: ENSG00000130303) was identified as a restriction factor of particular significance, as the ability to antagonise Tetherin appears to have been a major factor in the adaptation of SIVcpz to man [2]–[4].

As implied by its name, Tetherin has the ability to tether HIV particles to the surface of infected cells, and this function is attributable to its unusual topology. Tetherin contains two membrane anchors, an N-terminal transmembrane domain and a C-terminal GPI-anchor [5], [6]. During assembly and budding of HIV particles at the plasma membrane (PM) of infected cells, Tetherin can be incorporated into nascent virions via one of its membrane anchors, leaving the second anchor in the PM, and thereby preventing virus release into the extracellular milieu [7]–[9]. The failure to release free particles inhibits the cell-free spread of HIV, which relies on the diffusion of released virus toward its target cells.

HIV and related viruses have evolved mechanisms to overcome Tetherin restriction and ensure their efficient propagation. In the case of HIV-1 main (M) group viruses, the accessory protein Vpu enhances lysosomal sorting and degradation of Tetherin, thereby reducing the levels of the restriction factor at the cell surface, and promoting HIV-1 release [10]–[12].

In addition to cell-free transmission, HIV can be transferred across intercellular contacts. This cell-cell spread appears to be significantly more efficient than cell-free propagation, and has been proposed to occur via filopodial bridges, membrane nanotubes, and, most prominently, virological synapses (VS) [13]. VS between T cells are characterised by the recruitment of viral proteins and HIV receptors to the cellular interface [14], however, little is known about VS between HIV-infected macrophages and T cells [15], [16]. Moreover, whether Tetherin also inhibits cell-cell spread of HIV, or whether a direct contact between the infected and the target cell eliminates the need for HIV to fully detach from its host cell, remains controversial [17]–[23].

CD4^+^ T cells and macrophages are the main cellular targets of HIV *in vivo*. Significantly, some aspects of viral replication vary with the target cell type. A prominent example is the site of virus assembly: Whereas in T cells HIV assembles and buds at the cell surface, in monocyte-derived macrophages (MDM) budding intermediates are almost exclusively detected on deeply invaginated PM domains [24], [25], which we have termed Intracellular Plasma Membrane-connected Compartments (IPMC) [26]. IPMCs have a neutral pH [27] and contain numerous molecules typically found at the PM, including phosphatidylinositol-4,5-bisphosphate [28], the tetraspanins CD9 and CD81, the hyaluronan receptor CD44 [24], and focal adhesion proteins including the integrins CD11b, CD11c, and CD18 [26]. Though IPMCs are also present in uninfected MDMs, and thus not induced by infection, HIV triggers changes in both the composition and morphology of the compartments: For example, IPMCs in HIV-infected MDMs contain the tetraspanin CD63 [24] and may be larger than in uninfected cells [29].

Given these variations in HIV replication in different cell types, it is imperative to analyse the localisation, function and antagonism of Tetherin specifically in macrophages. This notion is re-enforced by a recent study suggesting important differences in Tetherin antagonism in macrophages and non-monocytic cells, namely that in MDMs Tetherin is only mildly induced by IFN, and that cell surface Tetherin is inefficiently antagonised by Vpu [21].

Here we show that Tetherin expression in primary MDMs is as sensitive to IFN as in primary T cells. At steady state, endogenous Tetherin localises to the cell surface, the *trans*-Golgi network (TGN), and IPMCs. Vpu efficiently antagonises cell surface Tetherin in MDMs and, in cells devoid of Vpu, Tetherin retains mature HIV particles in IPMCs, which may cause a virus particle-induced expansion of the assembly compartments. Furthermore, our experiments indicate that cell-cell transmission allows efficient spread of HIV from MDMs to autologous CD4^+^ T cells. Using a novel assay that strongly favours cell-cell over cell-free propagation, we show that Tetherin can restrict cell-cell transmission of HIV from macrophages to T cells. Thus, our data indicate that Tetherin has the potential to impose a major restriction to HIV spread, regardless of the mode of transmission.

## Results

### Type I interferons upregulate Tetherin expression in primary macrophages

**Figure 1:**
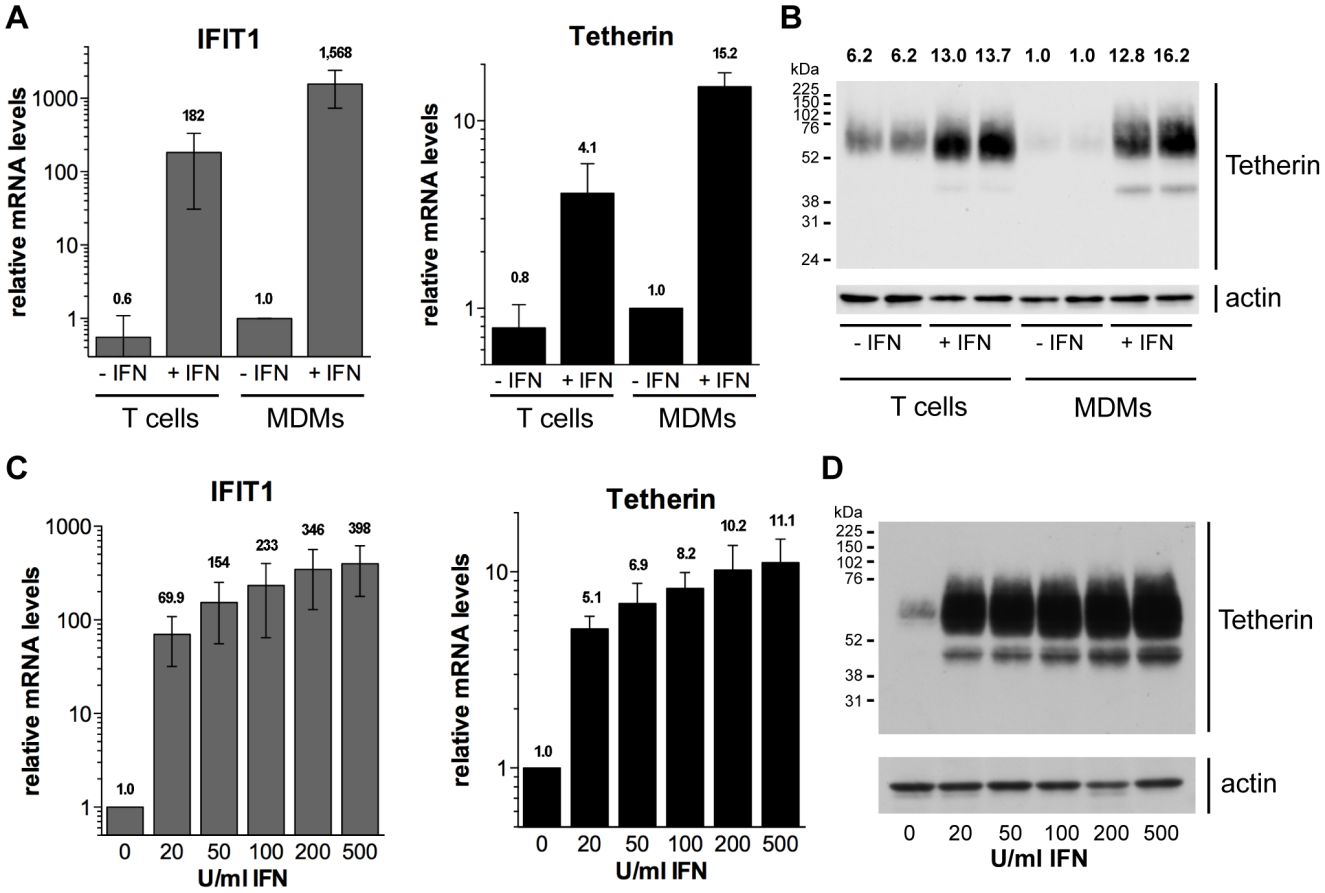
Type I interferons upregulate Tetherin expression in primary macrophages. (A–B) Primary MDMs and autologous lectin/IL-2-activated CD4^+^ T cells were stimulated for 24 h with 544 U/ml (2 ng/ml) IFN-β eight days post isolation from buffy coats. (A) IFIT1 and Tetherin mRNA levels were determined by RT-qPCR and normalised to GAPDH. Bars represent the means ± standard deviations (SD) from four donors relative to untreated MDMs (−IFN, set at 1). (B) Tetherin protein levels were determined by western blot analysis of whole cell lysates containing equal amounts of total protein. Numbers above each lane indicate the Tetherin band intensities relative to untreated MDMs (−IFN, set at 1). (C–D) MDMs were stimulated for 24 h with 0–500 U/ml IFN-β eight days post isolation from buffy coats. (C) mRNA levels were determined as described for (A). Bars represent the means ± SD from three donors relative to untreated MDMs (set at 1). (D) Tetherin protein levels were determined by western blot analysis of whole cell lysates.

Numerous studies have shown that type I IFNs induce the expression of Tetherin in cell lines and primary T cells, but whether this is also true for primary macrophages remains controversial [21], [30]. To determine whether type I IFNs induce endogenous Tetherin expression in macrophages, we isolated monocytes and CD4^+^ T cells from buffy coats of HIV-negative donors, differentiated the monocytes into MDMs *in vitro*, and proliferated the T cells in the presence of lectin and IL-2. Subsequently, all cells were stimulated with 544 U/ml (2 ng/ml) IFN-β for 24 h, and analysed by RT-qPCR and western blotting. Increased mRNA levels of the IFN-induced gene IFIT1 in both MDMs and T cells confirmed that the IFN-β preparation was biologically active and induced the IFN pathway (Fig. 1A). Notably, IFN-β treatment of MDMs upregulated both the mRNA and protein levels of Tetherin by approximately one order of magnitude (Fig. 1A,B). In autologous T cells, IFN-β treatment increased the Tetherin levels two- to five-fold (Fig. 1A,B).

We next examined the concentration dependence of the Tetherin upregulation by type I IFNs. Following treatment of MDMs with 20–500 U/ml IFN-β, we observed significant increases in both IFIT1 and Tetherin mRNA levels (Fig. 1C), as well as Tetherin protein levels (Fig. 1D), even at the lowest IFN-β concentration tested. Together our data demonstrate that primary MDMs upregulate Tetherin expression, even at low concentrations of a type I IFN, to an extent comparable to autologous CD4^+^ T cells.

### Tetherin localises to the cell surface, TGN, and IPMCs in MDMs

**Figure 2:**
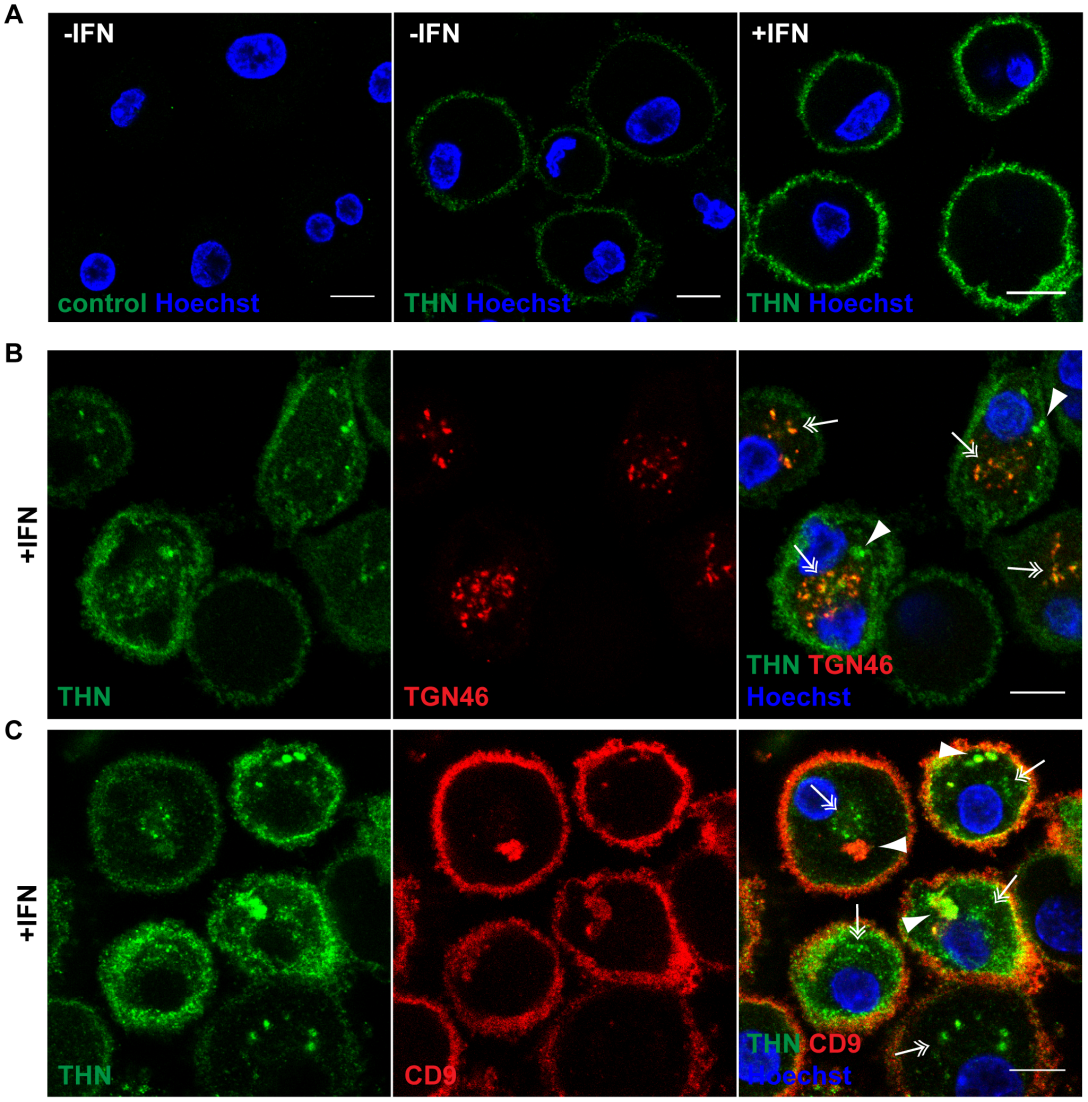
Tetherin localises to the cell surface, TGN, and IPMCs. (A) Untreated MDMs (−IFN), or MDMs treated for 24 h with 544 U/ml IFN-β (+IFN), were incubated for 1 h on ice in media containing 10 µg/ml polyclonal Tetherin (THN) antibody (B02P), or VSV-G antibody as a negative control. Cells were fixed and labelled with a fluorescent secondary antibody. (B–C) IFN-β-treated MDMs were incubated for 20 min on ice with 10 µg/ml polyclonal Tetherin antibody (B02P) and 2.5 µg/ml anti-TGN46, or with 10 µg/ml monoclonal Tetherin antibody (M15) and 2 µg/ml anti-CD9, in the presence of 0.05% saponin. Cells were fixed and labelled with fluorescent secondary antibodies. Arrowheads point at structures reminiscent of IPMCs, double arrows indicate TGN-like staining patterns. All images are single confocal sections. Scale bars = 10 µm.

We selected two commonly used, commercially available antibodies to examine the cellular distribution of endogenously expressed Tetherin in primary MDMs. Immunolabelling was performed on live cells, as all Tetherin antibodies tested exhibited reduced binding efficiency when applied after aldehyde fixation. When we incubated unpermeabilised MDMs in antibody-containing media on ice, then fixed and stained with a fluorescent secondary antibody, endogenous Tetherin was readily detected on the surface of primary MDMs (Fig. 2A). Consistent with an increase in mRNA and overall protein levels (Fig. 1), the levels of cell surface Tetherin were increased following IFN treatment (Fig. 2A).

We next permeabilised MDMs and incubated them with polyclonal antibodies against Tetherin and TGN46, or monoclonal antibodies against Tetherin and CD9, on ice. In both cases Tetherin was detected in two distinct intracellular locations: In almost all cells Tetherin was found in spots (double arrows in Fig. 2B,C), often distributed around the nucleus, which co-localised with TGN46 (double arrows in Fig. 2B). Some MDMs showed additional accumulations of Tetherin (arrowheads in Fig. 2B,C), which co-stained for the IPMC protein CD9 (arrowheads in Fig. 2C). Also in permeabilised MDMs, Tetherin levels were higher in IFN-stimulated than in untreated cells (compare Fig. 2B,C to Fig. S1A,B).

We conclude that in primary MDMs, endogenous Tetherin localises to the cell surface, TGN, and IPMCs, without any obvious enrichment of the protein in IPMCs. This is consistent with the notion that IPMCs are continuous with, and biochemically similar to, the PM [24], [25].

### Vpu efficiently antagonises Tetherin in MDMs

**Figure 3:**
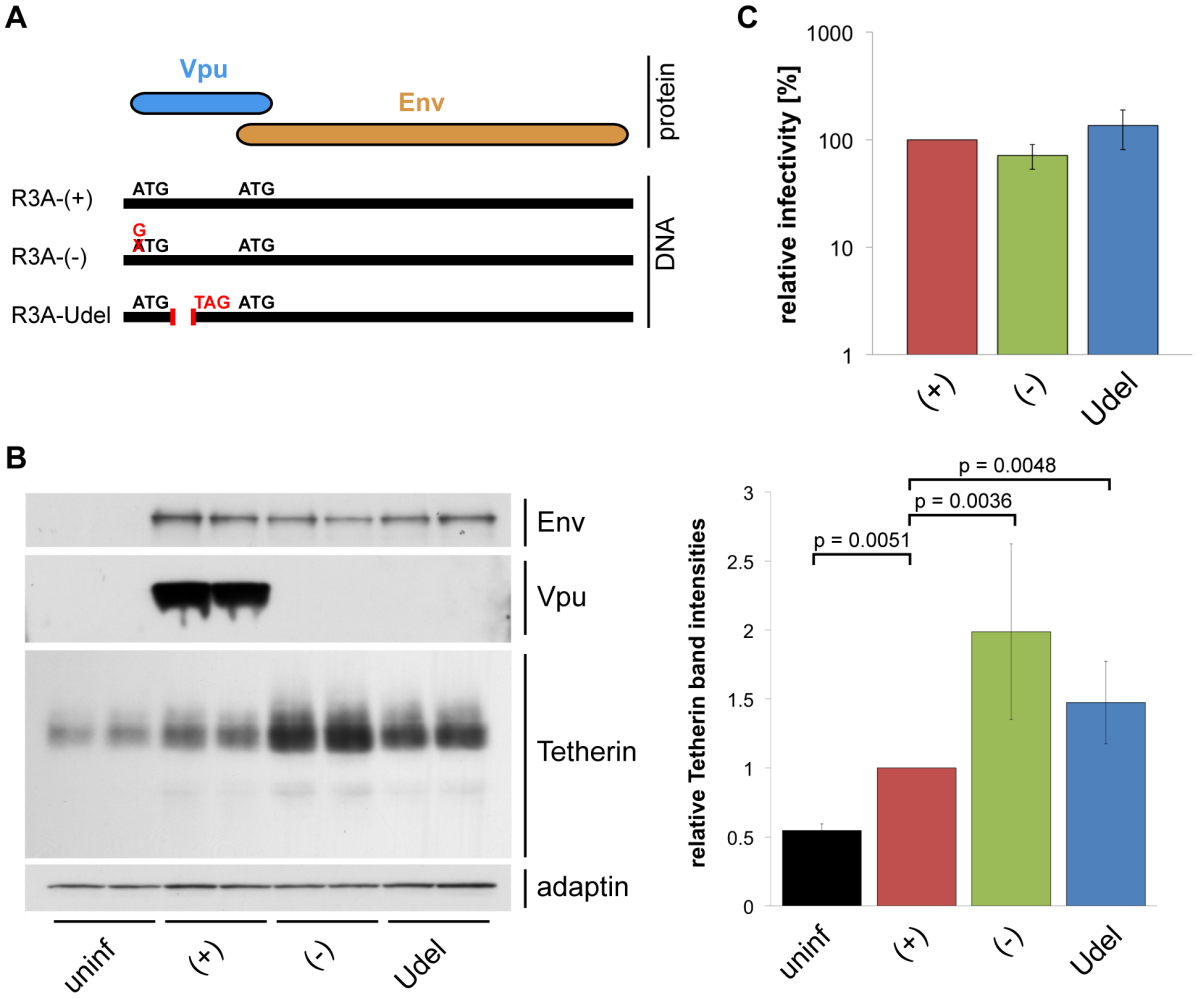
Vpu efficiently antagonises Tetherin in MDMs. (A) Schematic representation of the R3A molecular clones used in this study. R3A-(+) expresses a Vpu protein, R3A-(−) and -Udel do not. (B) MDMs were infected with R3A-(+), -(−), or -Udel for seven days, lysed, and analysed by western blotting. The Tetherin band intensities were quantified and normalised to the γ-adaptin levels, respectively. Bars represent the means ± SD of duplicate samples from three donors relative to R3A-(+) (set at 1). (C) Cell-free culture supernatants from R3A-infected MDMs were collected, p24 Gag levels adjusted, and single-cycle infectivities determined using TZM-bl cells. Bars represent the means ± SD of triplicate samples from four donors relative to R3A-(+) (set at 100%).

To investigate whether HIV-1 Vpu antagonises endogenous Tetherin in primary macrophages, we disrupted the Vpu gene of the dual-tropic HIV-1 strain NL4.3-R3A (from hereon referred to as R3A-(+)): R3A-(−) carries a start codon mutation, and R3A-Udel an internal deletion in Vpu (Fig. 3A). We used both Vpu mutants, R3A-(−) and -Udel, since MDMs infected with a Vpu start codon-deleted HIV-1 have been suggested to express increased levels of Env, which may affect Tetherin antagonism and viral infectivity [31]. Western blot analyses confirmed that Vpu was expressed in MDMs infected seven days after monocyte isolation with R3A-(+) for seven days, but not in cells infected with R3A-(−) or -Udel (Fig. 3B). Significant differences in Env expression were not detected (Fig. 3B) and, consistently, single-cycle infectivity assays indicated that MDM-derived R3A-(−) and -Udel were as infectious as R3A-(+) (Fig. 3C).

We performed western blot analyses to test whether Vpu antagonises Tetherin in primary MDMs. We found that Tetherin levels in MDMs infected with R3A-(+) for seven days were decreased compared to R3A-(−) and –Udel-infected cells (Fig. 3B), showing that Vpu reduces the overall levels of endogenous Tetherin in MDMs. The higher Tetherin levels detected in infected compared to uninfected MDMs were most likely due to a cellular IFN response. Consistently, western blotting showed that Tetherin levels in R3A-infected MDMs were reduced when we infected and cultured the cells in the presence of 1 µg/ml of an IFN-α/β receptor antibody that has been shown to prevent activation of the IFN receptor (Fig. S2, [32]).

**Figure 4:**
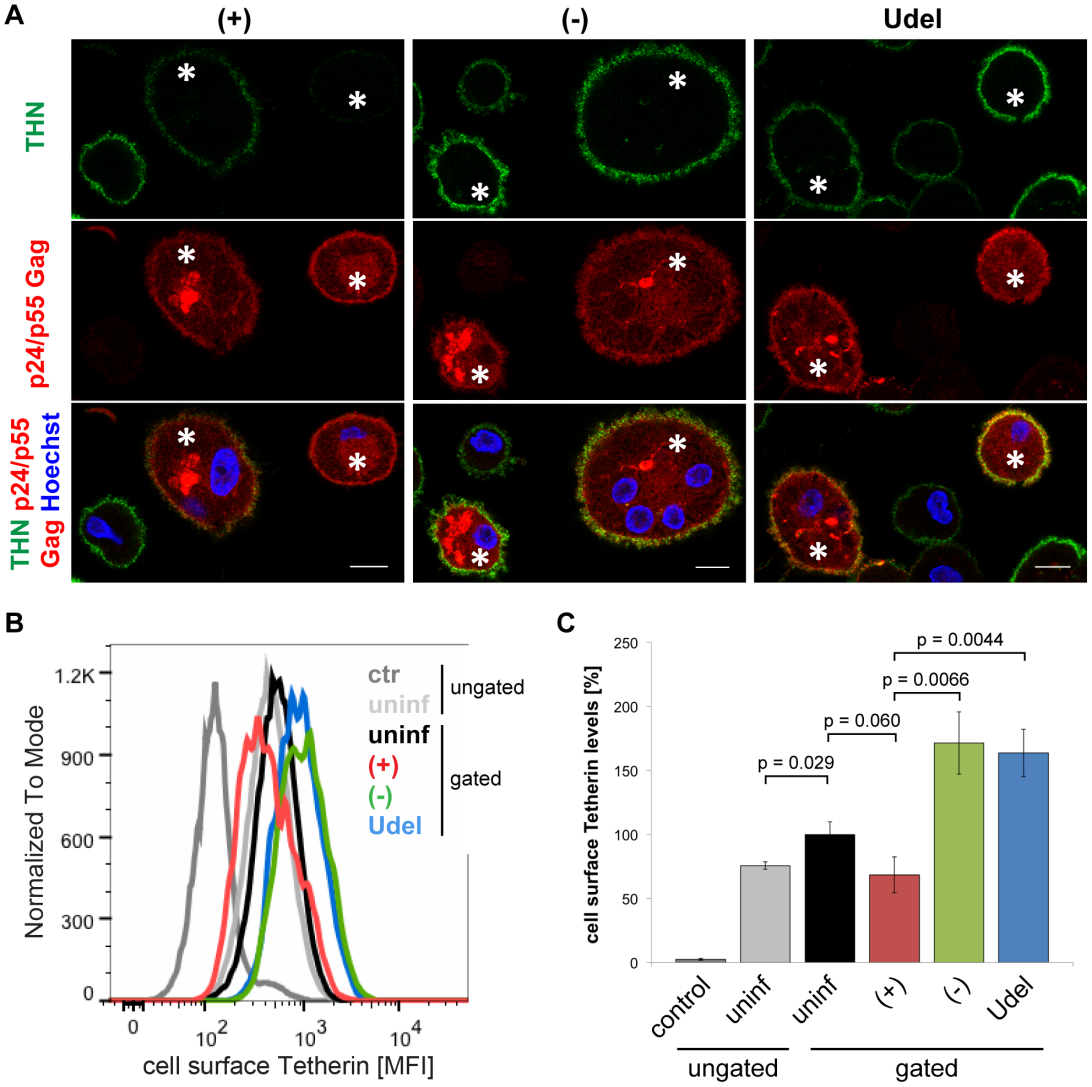
Vpu antagonises cell surface Tetherin in MDMs. (A) R3A-infected MDMs were incubated for 1 h on ice in media containing 10 µg/ml polyclonal Tetherin (THN) antibody (B02P). Cells were fixed, permeabilised, labelled with a p24/p55 Gag antiserum, and stained with fluorescent secondary antibodies. Asterisks mark infected cells. Single confocal sections are shown. Scale bars = 10 µm. (B–C) Cell surface Tetherin on uninfected and R3A-infected MDMs was labelled as described for (A), uninfected MDMs were incubated with VSV-G antibody as a negative control, and all cells were analysed by flow cytometry. Uninfected cell populations were left ungated, infected cell populations gated on the uninfected or infected subpopulations. (B) shows the results of a representative experiment, the bars in (C) represent the average Tetherin mean fluorescence intensities (MFI) ± SD from three donors relative to the gated, uninfected subpopulations (set at 100%).

To analyse the effects of Vpu on cell surface Tetherin in MDMs, we immunolabelled R3A-infected cells in media containing Tetherin antibody on ice. Following fixation, the MDMs were permeabilised and immunolabelled with a p24 Gag antiserum, which also recognises cytosolic p55 Gag and thus allows the unambiguous identification of infected cells. Compared to uninfected cells of the same populations, the levels of cell surface Tetherin were slightly reduced on R3A-(+)-infected, but significantly increased on R3A-(−) and -Udel-infected MDMs (Fig. 4A). Flow cytometry analyses confirmed these observations, with Tetherin levels on R3A-(−) and –Udel-infected MDMs at least two-fold higher than on R3A-(+)-infected cells (Fig. 4B,C). Interestingly, the cell surface Tetherin levels on uninfected MDMs within infected cell populations were higher than on completely untreated cells (Fig. 4B,C). These observations are consistent with our hypothesis that long-term HIV infection can trigger a cellular IFN response, which would lead to the secretion of IFNs and an upregulation of Tetherin in surrounding cells. A cellular IFN response could also explain the increased cell surface Tetherin levels seen on R3A-(−) and –Udel-infected compared to uninfected MDMs (Fig. 4B,C).

Together, our data show that Vpu reduces the overall levels of endogenous Tetherin in MDMs. The high efficiency of Tetherin labelling we achieved allowed us to also detect Vpu-induced downregulation of the restriction factor from the cell surface.

### Tetherin retains HIV in the IPMCs of MDMs

**Figure 5:**
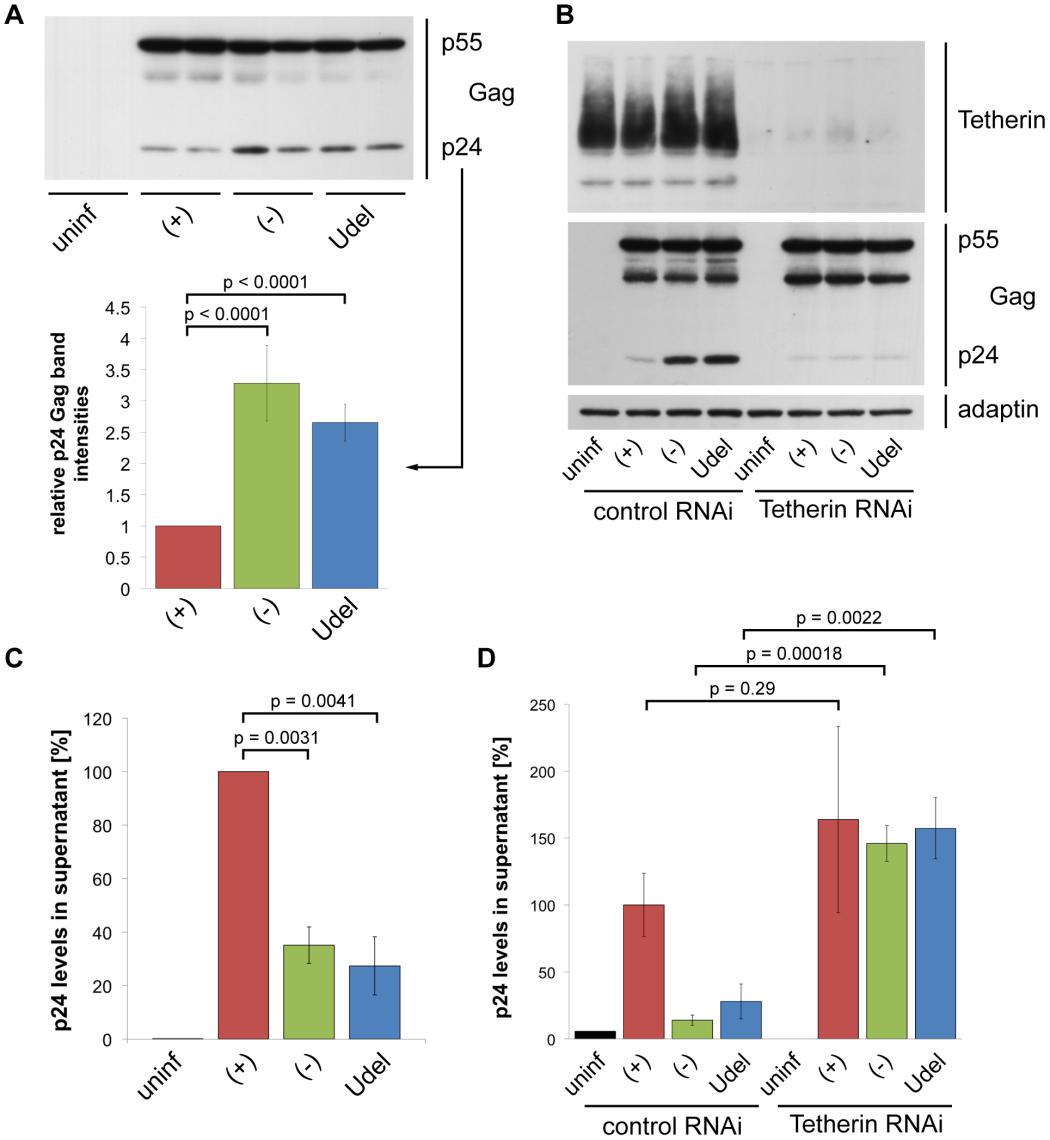
Tetherin retains HIV on infected MDMs. (A) MDMs were infected with R3A-(+), -(−), or -Udel for seven days, lysed, and analysed by western blotting. Note that the γ-adaptin blot from this donor is shown in Fig. 3B. The p24 Gag band intensities were quantified and normalised to the p55 Gag levels. Bars represent the means ± SD of duplicate samples from three donors relative to R3A-(+) (set at 1). (B) MDMs were infected with R3A and transfected with control or Tetherin siRNA the next day. Cells were lysed six days later and analysed by western blotting. (C) p24 Gag concentrations in cell-free culture supernatants from R3A-infected MDMs were determined using ELISAs. Bars represent the means ± SD of triplicate samples from three donors relative to R3A-(+) (set at 100%). p values were calculated using a paired Student's t-test before normalisation. (D) p24 Gag concentrations in cell-free culture supernatants from R3A-infected, siRNA-treated MDMs were determined using ELISAs. Bars represent the means ± SD of triplicate samples from a representative experiment, relative to control RNAi/R3A-(+) (set at 100%).

We next sought to examine if and where Tetherin retains HIV on primary macrophages. When we infected MDMs with R3A for seven days and performed western blot analyses of the cell lysates, we found significantly more p24 Gag associated with R3A-(−) and –Udel, than with R3A-(+)-infected cells (Fig. 5A). p24 and p17 Gag are predominantly found in mature HIV particles, maturation involving the cleavage of p55 Gag during or shortly after budding. The increased p24 Gag levels therefore indicated that, in the absence of Vpu, budded HIV particles were retained on MDMs, presumably by the elevated levels of Tetherin. Consistently, when we depleted MDMs of Tetherin by RNAi, only low levels of p24 Gag were associated with R3A-(+), -(−) and –Udel-infected cells (Fig. 5B).

To directly test whether Tetherin-mediated retention of virus impedes the release and thus cell-free spread of HIV from primary macrophages, we quantified virus released from R3A-(+), -(−), and –Udel infected MDMs. p24 ELISAs showed that HIV release into the supernatant was lower in the absence of Vpu than in its presence (Fig. 5C), and this restriction was overcome by depleting MDMs of Tetherin (Fig. 5D).

**Figure 6:**
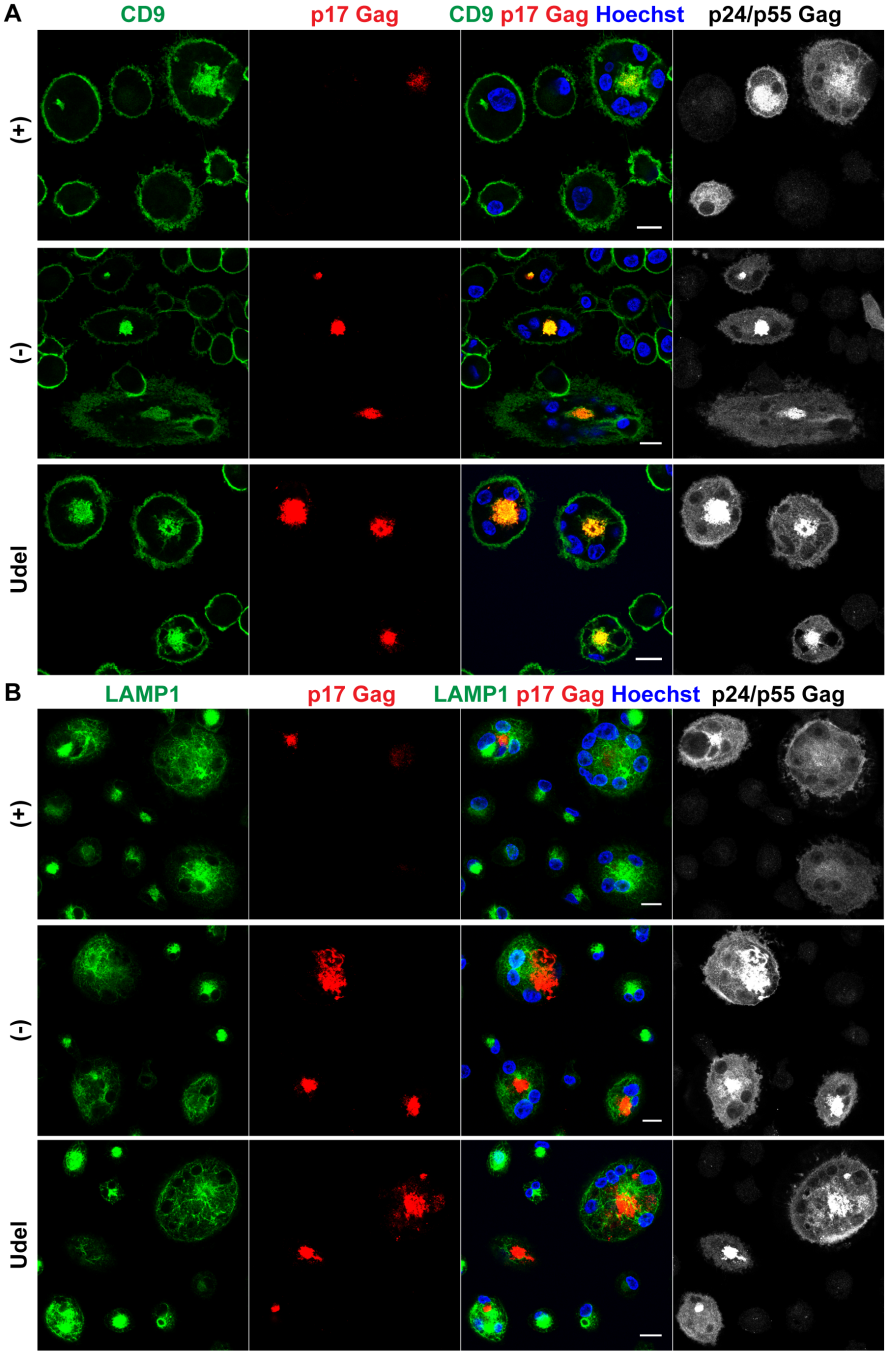
In the absence of Vpu, HIV accumulates in IPMCs. (A–B) MDMs were infected with R3A-(+),-(−), or -Udel for seven days, fixed, and immunostained for the indicated proteins. Single confocal sections are shown. Scale bars = 15 µm.

To examine the localisation of retained HIV particles, we immunostained R3A-infected MDMs with a p17 Gag antibody that specifically labels mature HIV particles, and a p24/p55 Gag antiserum to identify infected cells. Consistent with the western blot analyses, more p17 Gag was associated with R3A-(−) and –Udel than with R3A-(+)-infected MDMs (Fig. 6A,B), and quantification of this effect by flow cytometry revealed a two- to three-fold difference in the p17 Gag mean fluorescence intensities (Fig. S3). Most retained HIV was intracellular (Fig. 6A,B), and co-staining experiments showed that both in the presence and absence of Vpu the intracellular virus co-localised with the IPMC-enriched tetraspanin CD9 (Fig. 6A), but not with the lysosomal marker LAMP1 (Fig. 6B).

Thus, in the absence of Vpu, endogenous Tetherin retains mature HIV in the IPMCs of primary MDMs, and restricts cell-free viral spread.

### Tetherin is not required to maintain IPMCs

**Figure 7:**
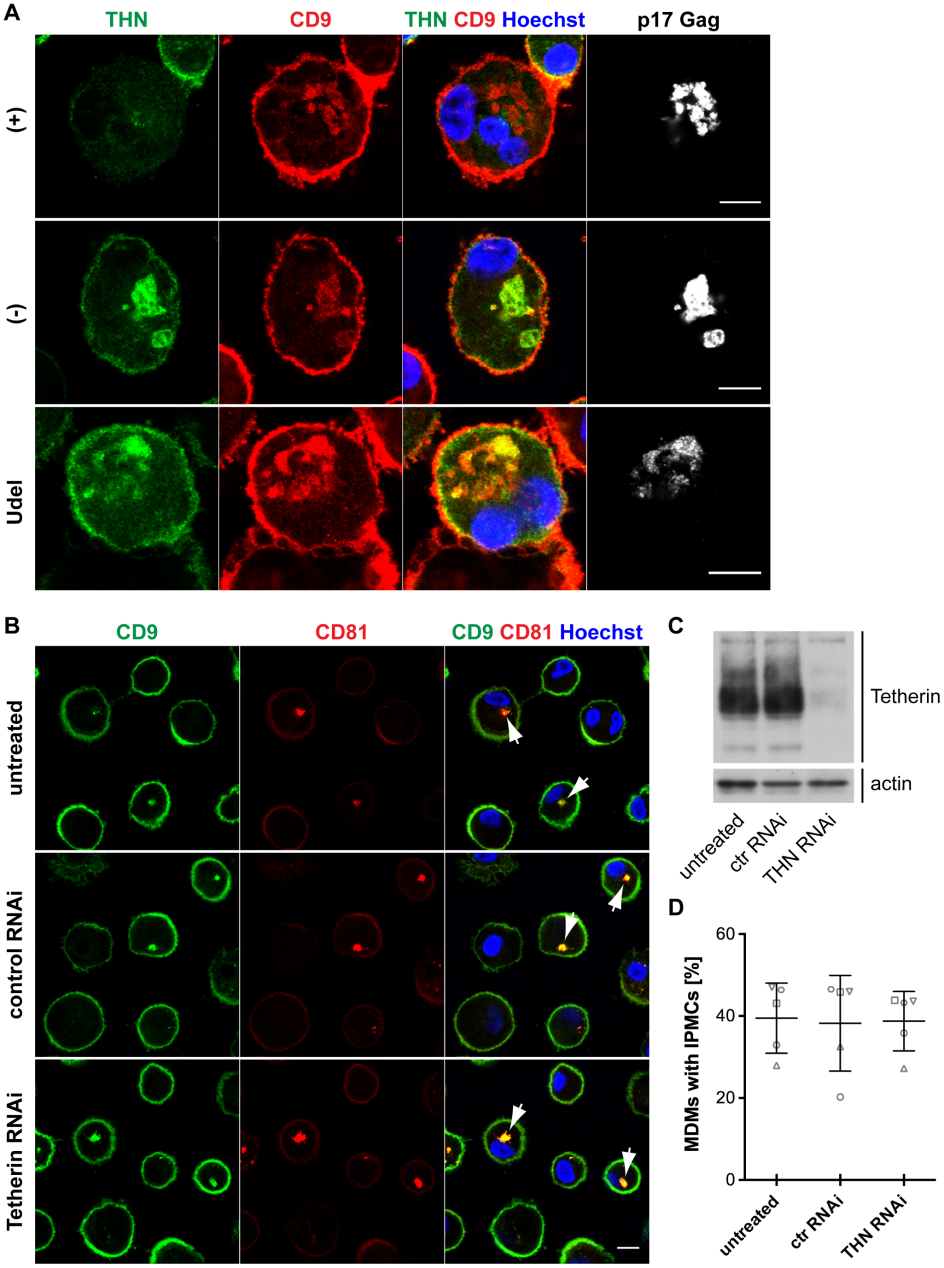
Tetherin is not required to maintain IPMCs. (A) IFN-β-treated MDMs were incubated for 20 min on ice with 10 µg/ml monoclonal Tetherin antibody (M15) and 2 µg/ml anti-CD9 in the presence of 0.05% saponin. Cells were fixed, labelled with anti-HIV-1 p17 Gag, and immunostained with fluorescent secondary antibodies in the presence of 0.1% Triton X-100. Scale bars = 10 µm. (B) MDMs were left untreated, or transfected with Tetherin or control siRNA for seven days. All cells were fixed and immunostained for CD9 and CD81. Arrowheads point at examples of structures identified as IPMCs. All images are single confocal sections. Scale bar = 10 µm. (C) Parallel cultures to the ones shown in (B) were lysed seven days after siRNA treatment and analysed by western blotting. (D) MDMs were treated as described for (B), and the proportion of cells showing any intracellular CD9/CD81 co-localisation was quantified on nine random confocal stacks for each condition. Between 255 and 407 cells were counted for each data point. The graphs show the means ± SD from five donors, and each donor is represented by differently shaped data points.

Tetherin-mediated retention of HIV in the IPMCs of MDMs likely leads to a passive expansion of the assembly compartments, but it is unclear whether the restriction factor is required for the integrity of IPMCs *per se*. When we co-stained R3A-infected MDMs for Tetherin, CD9 and p17 Gag, as expected, Tetherin was found to accumulate in the IPMCs of R3A-(−) and –Udel-infected cells (Fig. 7A). Tetherin levels in the IPMCs of R3A-(+)-infected cells were lower than in R3A-(−) or -Udel-infected MDMs, even when IPMCs of similar sizes, which contained similar amounts of virus, were compared (Fig. 7A). These observations indicated that Vpu reduces Tetherin levels also in IPMCs, but that the assembly compartments are maintained even at low concentrations of the restriction factor. Consistently, when we treated uninfected MDMs with Tetherin or control siRNA and quantified the proportion of cells with intracellular co-localisation of the IPMC proteins CD9 and CD81, around 40% of MDMs contained IPMCs regardless of the Tetherin levels (Fig. 7B–D). Overall, these data show that Tetherin is not required to maintain IPMCs in primary MDMs.

### HIV is transmitted from MDMs to T cells via virological synapses

To determine whether Tetherin restricts cell-cell spread of HIV from macrophages to autologous CD4^+^ T cells, we initially examined the mode, kinetics, and efficiency of the direct MDM-T cell transmission.

**Figure 8:**
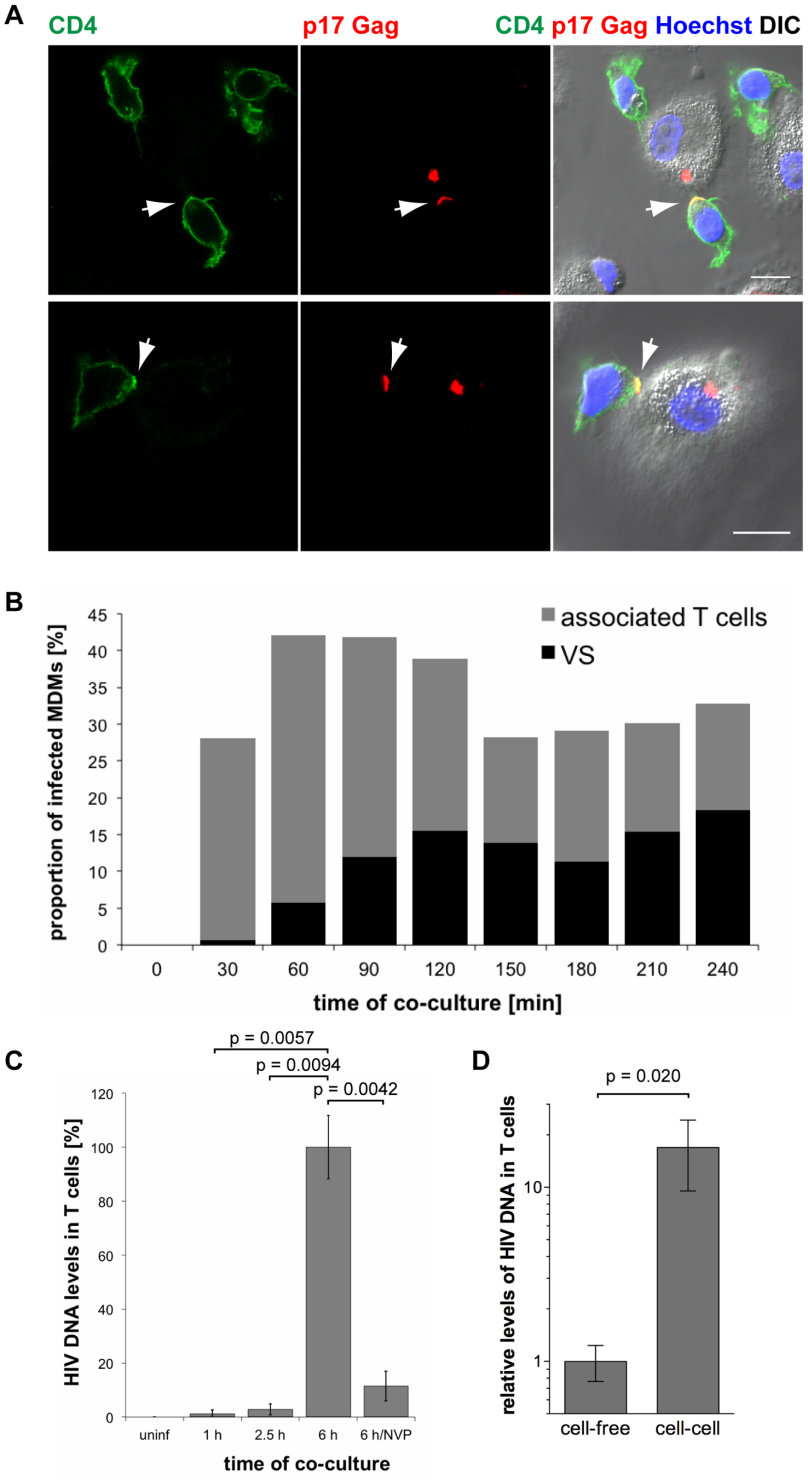
HIV is transmitted from MDMs to T cells via virological synapses. (A) MDMs were infected with HIV-1 BaL for seven days, co-cultured with autologous CD4^+^ T cells for 2.5 h, fixed and immunostained for the indicated proteins. Arrows indicate VS. Scale bars = 10 µm. (B) BaL-infected MDMs were co-cultured with autologous T cells for the indicated times, fixed, and immunostained for p17 Gag and CD4. No T cells were added to a control sample of MDMs (0 min). The proportion of infected MDMs that associated with T cells (black+grey bars), and formed VS (black bars) was counted on seven random confocal images with a total of 146–208 infected MDMs for each time point. (C) BaL-infected MDMs were co-cultured with autologous T cells for the indicated times, the T cells were washed off the MDMs, and Gag DNA levels in the T cells were quantified by qPCR. As controls, T cells were incubated for 6 h with uninfected MDMs (uninf), or with infected MDMs in the presence of 500 nM NVP (6 h/NVP). (D) Autologous CD4^+^ T cells were incubated for 6 h with BaL-infected MDMs, or with cell-free supernatants collected from the same MDMs during the preceding 6 h period. All T cells were collected and Gag DNA levels in the T cells quantified by qPCR. (C–D) Gag DNA levels were normalised to GAPDH. For cell-cell transmission experiments, the levels of contaminating MDM-derived Gag and GAPDH DNA were subtracted from the total DNA levels. Graphs show the means ± SD of triplicate samples from a representative experiment relative to 6 h (set at 100%), or cell-free (set at 1).

Immunofluorescence studies showed that primary CD4^+^ T cells readily associated with uninfected as well as HIV-1 BaL-infected MDMs within 2.5 h of co-culture (Fig. S4). When T cells were associated with infected MDMs, clusters of p17 Gag were occasionally found at the intercellular junctions, and in some cases CD4 co-clustered as well (Fig. 8A and Fig. S4). Junctions between infected MDMs and uninfected T cells characterised by an accumulation of mature HIV particles are from hereon referred to as virological synapses (VS). It has been suggested that MDM-T cell VS form by re-localisation of virus-filled IPMCs in MDMs to the MDM-T cell interface [16]. We observed that the IPMC proteins CD9 (Fig. S5A) and CD81 (Fig. S5B), as well as the β2 integrin CD18 (Fig. S5C), were also occasionally enriched at VS.

To determine the kinetics of MDM-T cell association and VS formation, we co-cultured BaL-infected MDMs with uninfected autologous CD4^+^ T cells, and fixed and immunostained the cells at 30 min intervals from 0 min (no T cells added) to 240 min. Though T cells rapidly associated with MDMs, few VS were detected after the first 30 min of co-culture (Fig. 8B). The proportion of infected MDMs with VS gradually increased between 30 and 120 min, and then remained relatively constant (Fig. 8B).

These observations suggested that the transfer of HIV from MDMs to T cells is mediated by VS. However, successful infection requires the fusion of viral particles with a target cell membrane, and reverse transcription of viral RNA genomes. We used qPCR to quantify the levels of HIV Gag DNA in T cells after co-culture with BaL-infected MDMs. Low levels of HIV DNA were detected in T cells after 1 and 2.5 h of co-culture (Fig. 8C), when VS formation had already peaked (Fig. 8B), but significantly higher levels were detected after 6 h (Fig. 8C). The reverse transcriptase inhibitor nevirapine (NVP) prevented HIV DNA accumulation, confirming that the qPCR assay detected only newly synthesised viral DNA (Fig. 8C).

We conclude that on co-culture of CD4^+^ T cells with autologous MDMs, T cell association with MDMs precedes VS formation, which in turn precedes the efficient MDM to T cell transfer of HIV, and T cell infection. This suggests that VS formation is an active process that plays a major role in the transmission of HIV from macrophages to T cells.

Finally, we determined the relative efficiencies of cell-cell and cell-free transmission of HIV. T cells co-cultured for 6 h with BaL- or R3A-infected MDMs contained at least ten times more HIV DNA than T cells incubated with cell-free virus released by the same MDMs during the preceding 6 h period (Fig. 8D and Fig. S6).

### Tetherin restricts cell-cell transmission of a dual-tropic HIV-1 from macrophages to T cells

**Figure 9:**
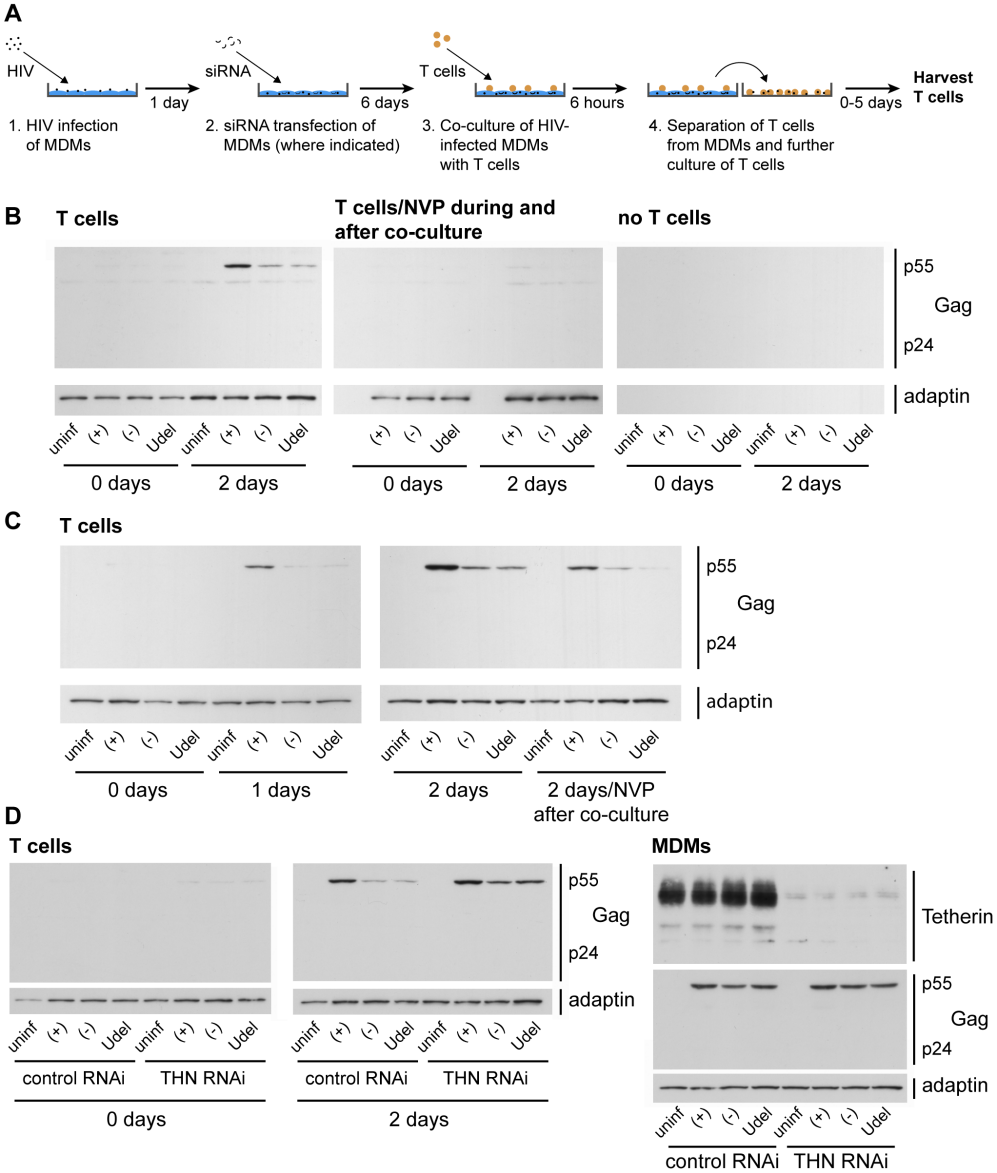
Tetherin restricts cell-cell transmission of a dual-tropic HIV-1 from MDMs to T cells. (A) Schematic outline of the assay used to quantify cell-cell transmission from MDMs to autologous CD4^+^ T cells. (B) R3A-(+), -(−), or -Udel-infected MDMs, or uninfected control MDMs, were co-cultured with autologous CD4^+^ T cells for 6 h, the T cells were washed off the MDMs with PBS and lysed immediately (0 days), or after another two day-incubation (2 days). As a control, 500 nM NVP was added to parallel MDM-T cell co-cultures, and to the T cells after the co-culture (T cells/NVP during and after co-culture). No T cells were added to infected MDMs, and the culture supernatants treated as the T cells, as another control (no T cells). All T cell lysates were analysed by western blotting. (C) T cells were co-cultured with R3A-infected MDM for 6 h, separated from the MDMs, and incubated for another zero to two days (0–2 days), or for two days in the presence of 500 nM NVP (2 days/NVP after co-culture). The T cells were lysed and analysed by western blotting. Note that parallel cultures of the MDMs used for the experiments in (B) and (C) were analysed by western blotting to confirm equal infection levels (Fig. S11). (D) MDMs were infected with R3A and transfected with control (control RNAi) or Tetherin (Tetherin RNAi) siRNA the next day. Six days later, autologous T cells were co-cultured with the infected MDMs for 6 h, separated from the MDMs, and lysed immediately (0 days) or incubated for another two days in the presence of 500 nM NVP (2 days). The T cell lysates (T cells), and lysates of the MDMs (MDMs) used for the experiment, were analysed by western blotting. Note that residual Tetherin levels in the MDMs had to be <5% to see a rescue of the T cell infection with R3A-(−) and -Udel.

Having shown that short-term co-cultures strongly favour cell-cell over cell-free HIV transmission (Fig. 8D and Fig. S6), we used this assay to study whether endogenous Tetherin can restrict the direct cell-cell spread of HIV from primary MDMs to autologous CD4^+^ T cells. Following 6 h of MDM/T cell co-culture, the T cells were washed off the MDMs with PBS, incubated further, and their overall infection assayed by western blotting (Fig. 9A). This approach allowed us to accurately determine the levels of T cell-associated p55 Gag, which is important when comparing the infection levels of cells that express, or do not express Vpu, as Tetherin alters the ratios of p24/p55 Gag (Fig. 5A). When we co-cultured T cells with R3A-(+), -(−), and –Udel-infected MDMs, and continued to incubate the T cells separately for two days, only the Vpu-containing R3A-(+) efficiently infected the T cells (Fig. 9B). This observation indicated that Tetherin can inhibit cell-cell transmission of HIV.

Control experiments were performed to confirm that the p55 Gag detected in the T cells after the co-culture was synthesised in these cells, and did not derive from the MDMs. As expected, no p55 Gag was detected in T cells that were harvested immediately after the co-culture with MDMs, or exposed to NVP during and after the co-culture, and no viral or cellular proteins were detected in the recovered media when the T cells were omitted (Fig. 9B). Further control experiments showed that Vpu expression in R3A-infected MDMs did not influence their adhesion to T cells (Fig. S7).

To demonstrate that Tetherin inhibits the transmission of HIV-1 from MDMs to T cells, and not the subsequent replication of the virus in T cells, we limited replication to a single round, either by adding NVP to the T cells immediately after the co-culture, or by harvesting the T cells after only one day. We still observed efficient infection of the T cells only with R3A-(+) (Fig. 9C). Notably, when we depleted R3A-infected MDMs of Tetherin before the co-culture (Fig. 9A), T cell infection with the Vpu-negative R3A-(−) and –Udel was rescued (Fig. 9D).

### Tetherin may restrict MDM-T cell transmission by promoting the transfer of HIV clusters

We next sought to investigate the mechanism by which Tetherin inhibits cell-cell transmission of HIV from macrophages to T cells. Hardly any p17 Gag accumulations were observed between R3A-infected MDMs and T cells, rendering us unable to quantify VS (Fig. S8A). We hypothesise that the p17 Gag accumulations at MDM-T cell interfaces are more transient when MDMs are infected with the dual-tropic R3A than with the CCR5-tropic BaL, as significantly more primary CD4^+^ T cells express CXCR4 than CCR5 at their surface (Fig. S8B,C), which may accelerate R3A entry into T cells.

**Figure 10:**
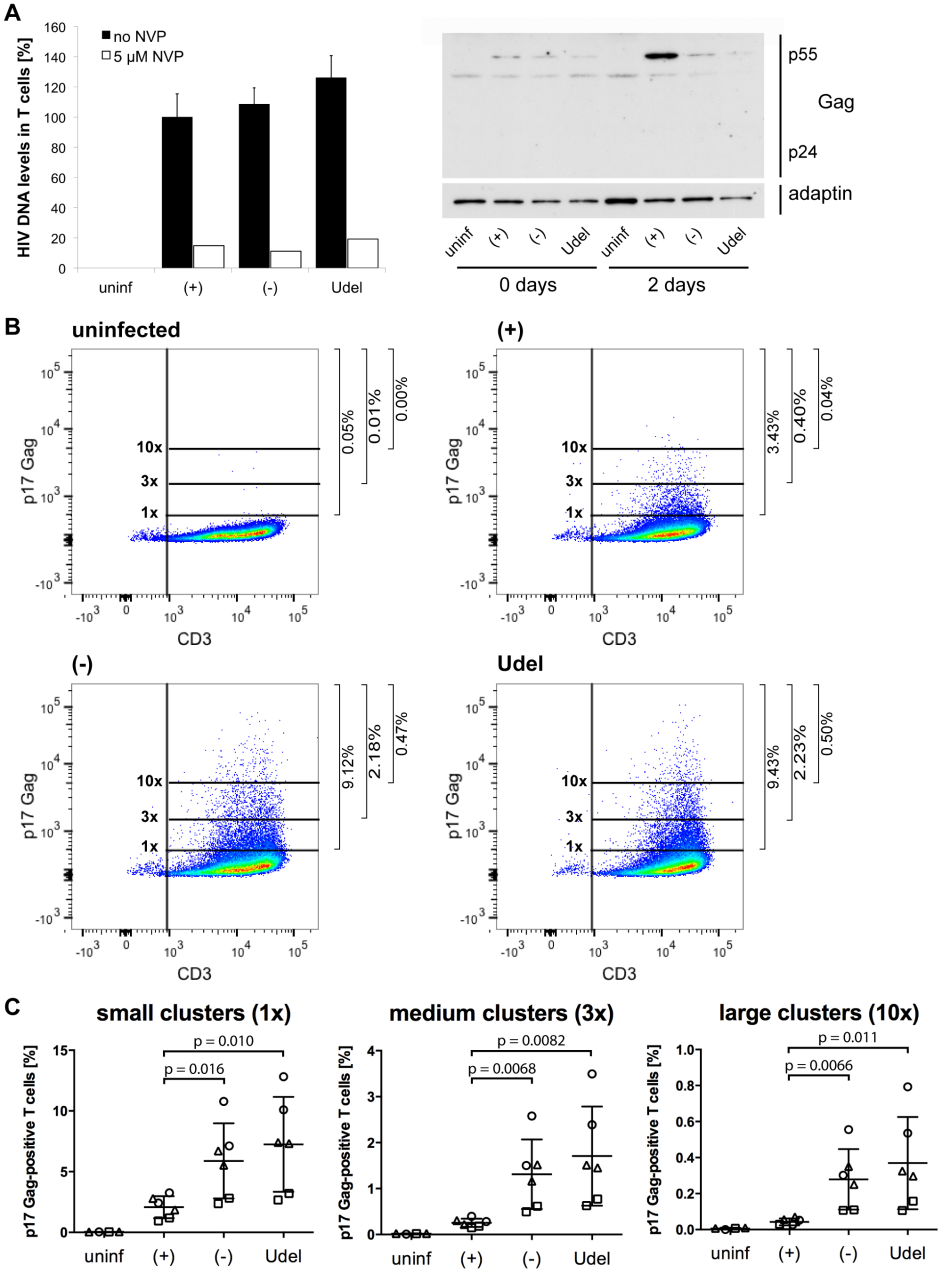
Tetherin promotes the transfer of infectious HIV clusters to T cells. (A) R3A-(+), -(−), or -Udel-infected MDMs, or uninfected control MDMs, were co-cultured with autologous CD4^+^ T cells for 6 h. As a control, 5 µM NVP was added to parallel MDM-T cell co-cultures. All T cells were separated from the MDMs, and aliquoted for qPCR and western blot analyses. For qPCR analysis, T cells were lysed immediately, and Gag DNA levels quantified by qPCR and normalised to GAPDH. The levels of contaminating MDM-derived Gag and GAPDH DNA were subtracted from the total DNA levels. Graphs for the “no NVP” samples show the means ± SD of triplicate samples from a representative experiment relative to R3A-(+) (set at 100%). Single values are shown for the “5 µM NVP” samples. For western blot analyses, T cells were lysed immediately (0 days), or after another two day-incubation (2 days). (B–C) T cells were co-cultured with R3A-infected MDMs for 6 h, separated from the MDMs, fixed, immunostained for p17 Gag and CD3, and analysed by flow cytometry. (B) shows the results of a representative experiment, and the numbers next to the gates indicate the proportions of cells with small (1×), medium (3×), and large (10×) p17 Gag clusters. (C) shows the mean proportions of cells with small (1×), medium (3×), and large (10×) p17 Gag clusters ± SD of duplicate samples from three donors, normalised to the MDM infection levels. Each donor is represented by differently shaped data points.

**Figure 11:**
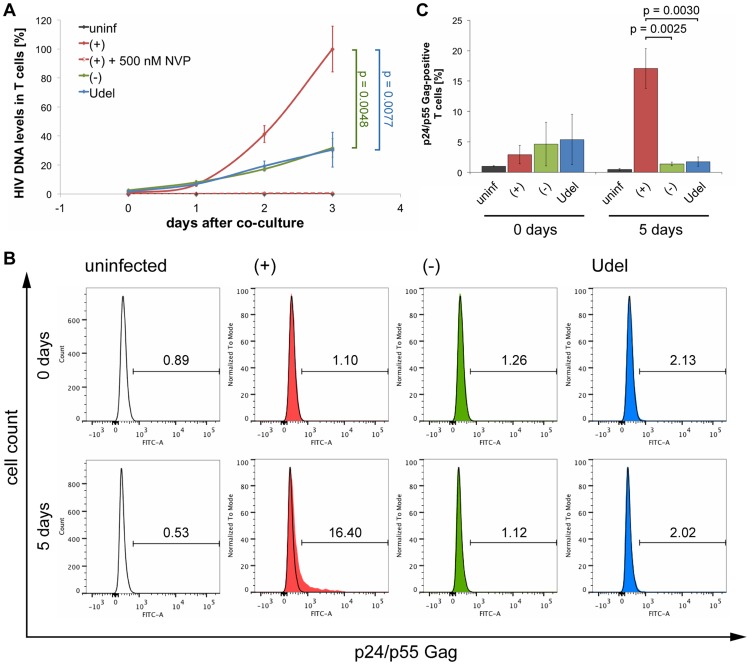
Tetherin can prevent MDMs from initiating a spreading infection in T cells. (A) R3A-(+), -(−), or -Udel-infected MDMs, or uninfected control MDMs, were co-cultured with autologous CD4^+^ T cells for 6 h, the T cells separated from the MDMs and lysed immediately (0 days), or after another one to three day-incubation (1–3 days). Gag DNA levels in the T cells were quantified by qPCR and normalised to GAPDH. As a control, 500 nM NVP was added to parallel MDM-T cell co-cultures, and to the T cells after the co-culture [(+)+500 nM NVP]. The levels of contaminating MDM-derived Gag and GAPDH DNA were subtracted from the total DNA levels. Graphs show the means ± SD of triplicate samples from a representative experiment relative to R3A-(+) after three days (set at 100%). (B–C) T cells were co-cultured with R3A-infected MDMs for 6 h, separated from the MDMs and fixed immediately (0 days), or incubated for another five days (5 days). The T cells were immunostained for p24/p55 Gag and CD3, and analysed by flow cytometry. (B) shows the results of a representative experiment, and the numbers above the gates indicate the proportions of Gag-positive cells. (C) shows the mean proportions of Gag-positive cells ± SD from three donors.

We next used Gag qPCR to investigate the early events of T cell infection following cell-cell transmission from MDMs. Intriguingly, similar levels of HIV DNA were detected in T cells immediately after their co-culture with R3A-(+), -(−) or -Udel-infected MDMs, although only R3A-(+)-infected T cells from the same experimental samples contained significant HIV protein levels two days later (Fig. 10A). Addition of NVP to the T cells during the co-culture inhibited the accumulation of HIV DNA (Fig. 10A). Since high Tetherin levels retain mature HIV particles on MDMs, we hypothesised that during cell-cell transmission in the absence of Vpu, infectious virus clusters may be transferred from MDMs to T cells, and lead to high HIV DNA levels in a few T cells, but overall a low proportion of infected cells. To test this hypothesis, we co-cultured R3A-(+), -(−), or –Udel-infected MDMs with T cells, immunostained the T cells for p17 Gag immediately after the co-culture and analysed them by flow cytometry. We found that T cells carried significantly more HIV clusters in the absence of Vpu than in its presence, and the difference was particularly pronounced for medium and large clusters (Fig. 10B,C). Moreover, when we incubated the T cells for longer times following their co-culture with MDMs, we found that R3A-(+) DNA accumulated much faster than R3A-(−) or –Udel DNA, and significantly higher R3A-(+) DNA levels were detected in the T cells after three days (Fig. 11A). Consistently, when we quantified the proportion of HIV-infected T cells by flow cytometry five days after their co-culture with infected MDMs, only T cells that had been co-cultured with R3A-(+)-infected MDMs contained a significant proportion of Gag-positive cells (Fig. 11B,C).

Together these data show that only in the presence of Vpu, do MDMs transmit sufficient HIV to initiate a spreading infection in T cells. In the absence of Vpu, endogenous Tetherin inhibits the cell-cell transmission of HIV from primary MDMs to autologous CD4^+^ T cells, presumably by promoting the transfer of infectious virus clusters to a limited number of target cells.

## Discussion

In recent years, an increasing number of cellular proteins that inhibit, or restrict, virus replication have been identified. Of these, Tetherin (HM1.24/CD317/Bst-2) stands out in that its efficient counteraction appears to have been crucial for the global spread of HIV [4]. Though SIV has crossed the species barrier from apes to man on at least four different occasions, giving rise to HIV-1 group M, N, O and P viruses, only group M HIVs, which have a fully functional antagonist of Tetherin, the Vpu protein, have spread globally [4], [33]–[35].

In the absence of an antagonist, Tetherin restricts cell-free propagation of HIV by physically linking mature virus particles to the surface of infected cells [2], [3], [7], [8]. Tetherin can also activate the NFκB signalling pathway, which may contribute to restriction [36]. A recent study showed that in addition to the full-length protein, cell lines and primary cells express a short isoform of Tetherin that lacks 12 N-terminal residues, is less sensitive to antagonism by Vpu, and cannot activate the NFκB pathway [37]. However, there is an increasingly strong view that direct cell-cell transmission of HIV is more efficient than cell-free propagation [13]. In this study we found that Tetherin can also restrict cell-cell transmission of HIV from macrophages to CD4^+^ T cells.

Macrophages, including neural microglia, are targets of HIV infection *in vivo* and, at least partially, responsible for HIV-associated dementia and neuropathy [38]. Several aspects of HIV replication in macrophages differ from other cell types: Whereas T cells are rapidly depleted early after HIV infection, macrophages appear to be more resistant to the cytopathic effects of HIV, and can survive for weeks to months following infection. This has led to the suggestion that macrophages may serve as reservoirs for HIV, particularly at the late stages of AIDS, when T cells are largely depleted [38].

Although both T cells and macrophages are major targets for HIV infection, the cell biology of virus replication in macrophages can differ to that seen in T cells. In infected tissue culture macrophages at least, the assembly of new virions is thought to occur predominantly in IPMCs (or virus containing compartments [VCC]), and not at the cell surface as seen in T cells [24], [25]. Some controversy exists as to whether IPMCs can transiently detach from the PM [39], but most data indicate that the majority of these compartments are contiguous with the cell surface [24], [25], [28]. IPMCs are thought to be largely impermeable to antibodies [40], [41]. *In vivo*, this may shield sites of HIV assembly from circulating neutralising antibodies, which may help HIV-infected macrophages to evade detection by the host immune system, and contribute to their long-term survival. Virus assembly and budding into IPMCs may also allow HIV release to be regulated, for example through VS [15], [16].

Because of the influence of Tetherin on the pandemic spread of HIV, the contribution of macrophages to HIV pathogenesis, and the cell type-specific differences in HIV replication, it is imperative to understand the effects of Tetherin on HIV replication in macrophages. To address this issue, we have relied almost entirely on primary human cells, i.e. macrophages derived from monocytes isolated from HIV-negative donors, and CD4^+^ T cells separated from the peripheral blood mononuclear cells of the same donors. This approach ensured that we studied cells expressing endogenous levels of Tetherin, thus avoiding possible effects of aberrant glycosylation and trafficking inherent to overexpression [7]. Moreover, we relied exclusively on HIV Env-mediated infection, avoiding possible artefacts that might result from the use of aberrant entry pathways and/or high multiplicities of infection of pseudotyped HIV.

By analysing both mRNA and protein, we found that type I IFNs upregulate the expression of Tetherin in MDMs even at low concentrations, and to an extent similar to that seen in IFN-treated T cells (Fig. 1). In the course of our study, we observed striking differences between western blots of Tetherin under non-reducing and reducing conditions: When we lysed MDMs in Laemmli buffer devoid of any reducing agent, Tetherin appeared as a prominent smear at ∼60–100 kDa and a weaker smear at ∼40 kDa (Fig. S9). These bands likely correspond to dimeric and monomeric forms of glycosylated Tetherin, respectively. Consistently, both bands completely disappeared upon Tetherin RNAi (Fig. 5B, 9D). Blotting the same lysates in the presence of 2-mercaptoethanol revealed a sharp, prominent band at ∼24 kDa, and a higher molecular weight smear appeared only with longer exposures of the blots (Fig. S9). We propose that the sharp band seen after reduction masks changes in Tetherin levels observed under non-reducing conditions, and may explain why a recent study found only moderate upregulation of Tetherin protein when treating MDMs with high levels of IFN [21].

Our immunofluorescence studies show that in uninfected MDMs, endogenous Tetherin localises to the cell surface, IPMCs, and TGN, without any obvious enrichment in IPMCs (Fig. 2). In cells infected with Vpu-deleted HIV, Tetherin retains virus in IPMCs (Fig. 6), which may result in a passive enrichment of Tetherin (Fig. 7A). As we know that accumulating virus expands IPMCs [29], tethered HIV will also cause a passive expansion of the size of the assembly compartments. These data, and the observation that less mature HIV is associated with MDMs following Tetherin RNAi (Fig. 5B), explain why a recent study detected smaller and fewer virus-filled IPMCs when depleting MDMs of Tetherin [21]. When investigated on uninfected MDMs, where IPMCs are not passively expanded by accumulating virus, we find no evidence that Tetherin plays an active role in the formation and/or maintenance of IPMCs (Fig. 7).

The improved Tetherin immunofluorescence labelling we achieved allowed us to detect cell surface Tetherin on MDMs, and Vpu-induced downregulation (Fig. 2A, Fig. 4), which was not observed in a recent study [21]. Low cell surface levels of Tetherin in the presence of Vpu corresponded with decreased overall levels (Fig. 3B). However, even in the presence of Vpu, HIV-infected MDM populations showed higher overall Tetherin levels than uninfected cells (Fig. 3B). We believe that this is caused by an IFN response to long-term HIV infection. Vpu would partially counteract the increased Tetherin expression in infected cells, but released IFN would lead to high Tetherin levels in the uninfected MDMs of the same population. In this situation, western blot analysis would show an increase in the overall Tetherin levels in the population (Fig. 3B), whereas flow cytometry, gated to the infected cells only, would detect decreased levels of cell surface Tetherin (Fig. 4B,C). Consistently, cell surface levels of Tetherin on uninfected cells within infected MDM populations were higher than on completely untreated cells (Fig. 4B,C), and Tetherin levels in R3A-infected MDMs decreased when we prevented activation of the IFN-α/β receptor using antibodies (Fig. S2). Nevertheless, our data show that HIV-1 Vpu efficiently antagonises endogenous Tetherin in primary macrophages. Residual Tetherin restriction may occur even in the presence of Vpu, as even less HIV was retained (Fig. 5B), and more released (Fig. 5D), upon Tetherin RNAi than in the presence of Vpu alone, but these differences were less pronounced and not statistically significant.

VS between infected and uninfected cells have been suggested to facilitate the cell-cell spread of HIV [13]. Although VS between infected macrophages and T cells have been observed [15], evidence that these are involved in HIV transmission is missing. In this study, we report that structures reminiscent of VS form between HIV-infected MDMs and autologous CD4^+^ T cells (Fig. 8A, Fig. S4). The temporal appearance of these VS is consistent with them mediating cell-cell transmission of virus, i.e. they succeed MDM-T cell interaction, but precede the appearance of HIV DNA in T cells (Fig. 8B,C). However, we cannot rule out the possibility that other modes of cell-cell transfer, including filopodial bridges and membrane nanotubes, may also contribute to HIV transmission from macrophages to T cells.

We have developed a novel co-culture assay to examine the effects of Tetherin on the cell-cell spread of HIV from infected MDMs to T cells (Fig. 9A). In contrast to other studies, in which infected and target cells were co-cultured for up to several days [18], [19], [21]–[23], we limited the co-culture to only 6 h. This approach prevented the accumulation of cell-free virus, and thus strongly favoured cell-cell transmission (Fig. 8D, Fig. S6). Our assay was sensitive enough to reliably detect even low levels of infection, as seen in our experiments as a result of the short co-culture and the use of primary cells. Finally, and again in contrast to other studies [17], [18], [21], [22], our assay allowed us to detect infection using the levels of p55 Gag only.

Using this assay, we found that Tetherin can inhibit cell-cell transmission of HIV from MDMs to autologous CD4^+^ T cells, and this effect was independent of whether or not the virus was allowed to replicate in the T cells (Fig. 9, Fig. 10, Fig. 11). On-going replication led to increasing levels of T cell infection at one and two days after their co-culture with R3A-(+)-infected MDMs, and intermediate infection levels were detected when NVP was added to the T cells for the two day-incubation after the co-culture. However, efficient T cell infection always depended on the downregulation of Tetherin in the MDMs, either by Vpu (Fig. 9B, C), or by RNAi (Fig. 9D).

Immediately after their co-culture with R3A-(+), -(−) or –Udel-infected MDMs, all T cells contained similar levels of HIV DNA, but only in the presence of Vpu was significant infection detected two days later (Fig. 10A). We hypothesise that, in the absence of Vpu, high Tetherin levels on MDMs promote the transfer of infectious clusters of HIV to T cells, which lead to high HIV DNA levels in a few T cells, but overall a low proportion of infected cells. Consistently, immediately after their co-culture with infected MDMs, we detected more and larger HIV clusters on T cells in the absence of Vpu than in its presence (Fig. 10B,C), and when HIV DNA and protein levels were assessed three or five days later, respectively, significantly more HIV DNA and a higher proportion of infected T cells were detected when Tetherin was antagonised by Vpu (Fig. 11). These observations are consistent with a previous study, which showed that in the absence of Vpu, HIV clusters are transferred from infected to uninfected T cells, but fail to initiate productive infection [17]. Notably, similar DNA levels in T cells immediately after their co-culture with infected MDMs confirmed that R3A-(−) and -Udel are as infectious as R3A-(+).

Recent evidence suggests that an accumulation of HIV DNA in activated or resting T cells may trigger innate immune responses, and lead to cell death by apoptosis or pyroptosis, respectively [42]–[44]. Therefore, during macrophage-T cell transmission of Vpu-deficient HIV, the accumulation of viral DNA in target cells may promote cell death, which could contribute to inefficient T cell infection. However, when we labelled T cells with a dead cell stain 0, 6, 18, and 30 h after their co-culture with HIV-infected MDMs, we did not detect increased T cell death in the absence of Vpu (Fig. S10).

*In vitro* at least, cell-cell transmission of HIV is thought to be more efficient than cell-free propagation. The high evolutionary pressure on SIV/HIV to maintain a Tetherin antagonist suggests that Tetherin inhibits both the cell-cell and cell-free spread of HIV. Although our data are consistent with this notion, there may be cell type-specific differences. For example, VS between T cells are thought to involve polarised budding of HIV into the synaptic cleft [14], whereas VS between monocytic cells and T cells may form by re-localisation of virus-filled IPMCs to the site of VS formation [16]. HIV that accumulates in IPMCs before reaching the VS may be more susceptible to clustering by Tetherin than newly budded virions in the T cell-T cell synapse. Consistently, most studies using monocytic cells, i.e. MDMs and monocyte-derived dendritic cells, found that Tetherin restricts cell-cell transmission of HIV [21], [22]. Similarly, Vpu-deficient HIV-1, as well as virus strains encoding mutated Vpu proteins, have been shown to inefficiently spread in macrophage populations [45].

Whether Tetherin also inhibits T cell-T cell spread remains controversial. A recent study suggested that Tetherin increases the number of VS formed between T cells, and thereby enhances target cell infection [18]. Consistently, in a previous study a Vpu-deficient HIV-1 clone emerged during selection of viruses that efficiently spread in a rapid-turnover culture of T cells [46]. However, other studies argue that Tetherin restricts the direct T cell-T cell transmission of HIV. In one study, clusters of Vpu-deficient HIV particles were seen to be transferred from infected to uninfected cells, but impaired in their ability to fuse with and thus infect target cells [17]. Still, Tetherin did not seem to perturb the formation of VS [17].

Overall our study shows that in MDMs Tetherin is upregulated even by low concentrations of type I IFNs, and localises to the cell surface, TGN, and IPMCs. Vpu efficiently antagonises Tetherin and, in the absence of Vpu, mature HIV accumulates in IPMCs. Although Tetherin-bound virus may expand IPMCs, there is no indication that Tetherin plays an active role in the formation and/or maintenance of the HIV assembly compartments. Finally, we find that Tetherin can restrict cell-cell transmission of HIV from MDMs to T cells, and the assay applied in this study may help elucidate whether the restriction factor also inhibits transmission between other cell types. Thus, this study provides crucial insight into one of the most potent HIV restriction factors identified to date, in one of the main target cells for HIV infection.

## Materials and Methods

### Reagents and antibodies

Tissue culture media and supplements were purchased from Life Technologies (Paisley, UK), Fetal Calf Serum (FCS) Gold from PAA (Yeovil, UK), human AB serum from PAA and Sigma-Aldrich (Dorset, UK), tissue culture plastic from Thermo Fisher Scientific (Waltham, USA) and TPP (Trasadingen, Switzerland), and chemicals from Sigma-Aldrich, unless specified otherwise. IFN-β was provided by M. Noursadeghi (UCL, London, UK), and nevirapine obtained from the AIDS Research and Reference Reagent Program (NIAID, Bethesda, USA).

Antibodies to CD4 (Q4120), HIV-1 p24/p55 Gag (38:96K and EF7) and HIV-1 p17 Gag (4C9), as well as an antiserum to HIV-1 p24/p55 Gag (ARP432), were obtained from the NIBSC Centre for AIDS Reagents (South Mimms, UK). Anti-CD68 (KP1) was provided by R. da Silva (University of Oxford, Oxford, UK), anti-CD81 (M38) by F. Berditchevski (University of Birmingham, Birmingham, UK), anti-CD81 (1.337) by J. Grove (UCL, London, UK), anti-CXCR4-Alexa Fluor 488 (12G5) by J. Hoxie (UPenn, Philadelphia, USA), anti-VSV-G (P5D4) by T. Kreis (UNIGE, Geneva, Switzerland), and antisera to Env gp120, HIV-1 p17 Gag (UP595) and Vpu (U2-3) by N. Haigwood (OHSU, Portland, USA), M. Malim (KCL, London, UK) and K. Strebel (NIAID, Bethesda, USA), respectively. Anti-HIV-1 p24/p55 Gag (Kal-1) was purchased from Dako (Ely, UK), anti-CD9 (MCA469G) and anti-TGN46 (AHP500G) from AbD Serotec (Kidlington, UK), anti-CD18 (MEM-48) and anti-CD3 (MEM-57) from Abcam (Cambridge, UK), anti-γ-adaptin (88/Adaptin γ), anti-CD3-PerCP (SK7), anti-CD3 (UCHT1), anti-CD4-FITC (RPA-T4), anti-CD195-PE (2D7/CCR5), anti-CD14-APC (M5E2) and anti-LAMP-1 (H4A3) from BD Biosciences (Oxford, UK), anti-actin (I-19) from Santa Cruz Biotechnology (Santa Cruz, USA), anti-IFN-α/β R2 (MMHAR-2) and mouse IgG2A isotype control (20102) from R&D Systems (Abingdon, UK), monoclonal and polyclonal anti-Bst-2 (M15 and B02P, respectively) from Abnova (Taipei, Taiwan), Alexa Fluor-conjugated antibodies from Life Technologies, and HRP-conjugated antibodies from Thermo Fisher Scientific.

### Plasmids and virus stocks

The Nef-negative HIV-1 molecular clone NL4.3-R3A, here referred to as R3A-(+), was provided by J. Hoxie (UPenn, Philadelphia, USA) [47], and used to avoid Nef-specific effects on Tetherin. To obtain R3A-(−), the Vpu start codon of R3A-(+) was mutated using the primers 5′-CTCTC TATCA AAGCA GTAAG TAGTA CATGT AGTGC AATCT TTACA AATAT-3′ and 5′-ATATT TGTAA AGATT GCACT ACATG TACTA CTTAC TGCTT TGATA GAGAG-3′ and the QuikChange II XL Site-Directed Mutagenesis Kit (Agilent Technologies, Wokingham, UK) according to the manufacturer's instructions. To obtain R3A-Udel, two unique XbaI restriction sites were introduced into the Vpu gene of R3A-(+) using the primers 5′-GTAAG TAGTA CATGT AATGC AATCT TTACA AATTC TAGAA ATAGT AGCAT TAGTA GTAGC AGC-3′ and 5′-GCTGC TACTA CTAAT GCTAC TATTT CTAGA ATTTG TAAAG ATTGC ATTAC ATGTA CTACT TAC-3′, and 5′-GTATG GTCCA TAGCA CTCAT AGAAT ATAGG AAAAT ATCTA GACAA AGAAA AATAG ACA-3′ and 5′-TGTCT ATTTT TCTTT GTCTA GATAT TTTCC TATAT TCTAT GAGTG CTATG GACCA TAC-3′, and the QuikChange II XL kit as described above. The resulting plasmid was digested with XbaI (Promega, Southampton, UK) and re-ligated without the 82 bp fragment of Vpu.

To produce virus stocks from molecular clones, HEK 293T cells were transfected with proviral DNA using FuGENE HD (Promega). Culture supernatants were harvested after two days and cleared of cell debris by centrifugation and filtration (0.45 µm). Viruses were pelleted through 25% sucrose cushions for 2 h at 100,000 *g* and 4°C and resuspended in complete medium (RPMI 1640, 100 U/ml penicillin, 0.1 mg/ml streptomycin, and 10% human AB serum). Stocks of HIV-1 BaL were prepared as described previously [24].

### p24 ELISA assay

p24 levels in cell-free supernatants from HIV-1-infected cells were quantified using the HIV-1 p24^CA^ Antigen Capture Assay Kit (AIDS and Cancer Virus Program, National Cancer Institute, Frederick, USA), or the QuickTiter HIV Lentivirus Quantitation Kit (Source BioScience, Nottingham, UK), according to the manufacturers' instructions.

### Single-cycle infectivity assay

The single-cycle infectivities of virus stocks were determined using TZM-bl indicator cells (provided by J. Martin-Serrano, KCL, London, UK). Cells were infected for 2 h at 1,300 *g* with dilutions of virus stocks containing 0.5–2 ng of p24 Gag or reference virus of known titres. β-galactosidase expression was quantified 24 h later using the Galacto-Star β-Galactosidase Reporter Gene Assay System (Life Technologies) and a PHERAstar Plus microplate reader (BMG LABTECH, Aylesbury, UK). Single-cycle infectivities of MDM-derived R3A were determined by incubating TZM-bl indicator cells with cell-free virus-containing culture supernatants containing 2–5 ng of p24 Gag and quantifying β-galactosidase expression 24 h later as described above.

### Cells and infections

MDMs were prepared from peripheral blood mononuclear cells (PBMC), isolated from buffy coats from HIV-negative blood donors (National Blood Service, Essex, UK), as described previously [48], and differentiated in complete medium containing 10 ng/ml of M-CSF (R&D Systems, Abingdon, UK) for two days. Where indicated, seven day-old MDMs were infected with HIV-1 (3 MOI/cell) by spinoculation for 2 h at 1,300 *g* and cultured for a further seven days. Unless specified otherwise, the MDMs were used after 14 days in culture.

To obtain autologous CD4^+^ T cells, the non-adherent fraction of the PBMCs was frozen and defrosted after nine days, unless specified otherwise. The cells were activated for three days with 1 µg/ml lectin from *Phaseolus vulgaris* (Sigma-Aldrich) and 5 ng/ml IL-2 (R&D Systems) in complete medium. CD4^+^ T cells were isolated using the CD4^+^ T Cell Isolation Kit (Miltenyi Biotec, Bisley, UK) according to the manufacturer's instructions, and cultured for a further two days in complete medium containing 5 ng/ml IL-2.

For co-cultures of MDMs and autologous CD4^+^ T cells, 3 T cells/MDM were added to the MDMs in complete medium and, unless specified otherwise, incubated for 6 h at 37°C and 5% CO_2_. The T cells were separated from the MDMs, residual T cells washed off with PBS, and all T cells lysed immediately or cultured in complete medium containing 5 ng/ml IL-2.

### Tetherin RNAi

MDMs were transfected with 60 nM Stealth siRNA targeting Tetherin (oligo ID HSS101115, Life Technologies), or Stealth siRNA Negative Control Med GC (Life Technologies), using Lipofectamine RNAiMAX (Life Technologies) according to the manufacturer's instructions. Transfection complexes were removed one day after transfection.

### DNA/RNA isolations, reverse transcription and quantitative PCR

Total DNA was isolated from T cells using the DNeasy Blood and Tissue Kit (QIAGEN, Manchester, UK) according to the manufacturer's instructions. 20–40 ng of DNA were used to quantify the levels of Gag and GAPDH using 500 nM of the previously characterised primers 5′-ACATC AAGCA GCCAT GCAAA T-3′ and 5′-ATCTG GCCTG GTGCA ATAGG-3′, and 5′-ACCAC AGTCC ATGCA TCACT-3′ and 5′-GGCCA TCACG CCACA GITT-3′ [49], respectively, the DyNAmo Flash SYBR Green qPCR Kit (Thermo Fisher Scientific), and a Mastercycler ep realplex 2 (Eppendorf, Stevenage, UK) with the following programme: 95°C for 7 min, and 40 cycles at 95°C for 10 s and 65°C for 30 s. Serial dilutions from one experimental sample were prepared for the standard curve. For MDM-T cell co-culture experiments, the levels of contaminating MDM-derived Gag and GAPDH DNA were subtracted from the total DNA levels. To determine the levels of MDM-derived DNA, medium only was added to HIV-infected MDMs and subsequently treated as the co-cultured T cells. MDM-derived Gag DNA levels were typically between 5 and 27% of the total levels for HIV-1 BaL, and between 5 and 17% for HIV-1 R3A. MDM-derived GAPDH levels were max. 2% of the total levels. Gag DNA levels were normalised to GAPDH.

Total RNA was isolated from cells using the RNeasy Plus Mini Kit (QIAGEN), and 50 ng of RNA reverse transcribed using the QuantiTect Reverse Transcription Kit (QIAGEN) according to the manufacturer's instructions. qPCR was performed as described above using 200 nM of IFIT1 and GAPDH primers from the IFNr qRT-Primer Set (Source BioScience), 200 nM of Bst-2 primers from the RT^2^ qPCR Primer Assay (QIAGEN), and the following cycler programme: 95°C for 10 min, 40 cycles at 95°C for 15 s, 60°C for 30 s and 72°C for 30 s, and 72°C for 10 min. No reverse transcriptase was added to control samples to confirm the complete elimination of genomic DNA. IFIT1 and Bst-2 RNA levels were normalised to GAPDH.

### Western blot analysis

For western blot analysis cells were washed in PBS and lysed in Laemmli Sample Buffer (Sigma-Aldrich) for 10 min at 95°C. The lysates were separated on SDS-polyacrylamide gels and transferred to Immobilon-P PVDF membranes (Millipore, Watford, UK) at 20 V for 1 h under semi-dry blotting conditions. Blots were quenched in 0.1% Tween/5% non-fat milk/PBS for 1 h at room temperature, incubated with primary antibody at 4°C overnight, washed three times with 0.1% Tween/PBS, and incubated with the appropriate HRP-conjugated secondary antibody for 1 h at room temperature. After five washes with 0.1% Tween/PBS, membranes were briefly incubated in SuperSignal West Pico/Dura/Femto Chemiluminescent Substrate (Thermo Fisher Scientific) and signals detected with Amersham Hyperfilm ECL (GE Healthcare Life Sciences, Little Chalfont, UK). For the comparison of Tetherin levels in T cells and MDMs, cells were lysed in non-reducing Laemmli buffer without bromophenol blue (10% SDS, 15% glycerol, 0.2 M Tris-HCl pH 6.8), total protein concentrations determined using the Bio-Rad DC Protein Assay (Bio-Rad, Hemel Hempstead, UK) according to the manufacturer's instructions, and 5 µg protein used for western blot analysis as described above. All Tetherin blots were performed under non-reducing conditions and using the polyclonal Bst-2 antibody B02P. Blots were scanned and quantified with Fiji.

### Immunofluorescence

For immunofluorescence, MDMs were washed with PBS, fixed in 4% PFA, quenched with 50 mM NH_4_Cl and permeabilised with 0.1% Triton X-100/0.5% BSA/6 µg/ml human IgG/PBS. Cells were labelled for 1.5 h with primary antibodies diluted in 0.5% BSA/6 µg/ml human IgG/PBS, washed in 0.5% BSA/PBS and incubated for 1 h with appropriate combinations of fluorescent secondary antibodies. Samples were washed, DNA stained with 10 µg/ml Hoechst 33258 in PBS, and coverslips mounted in Mowiol. Confocal images were acquired with an inverted Leica TCS SP5 confocal microscope, 63× oil objective (NA 1.4) and *LAS AF* software, and processed using Fiji. Where indicated live, unpermeabilised MDMs were incubated for 1 h on ice in complete medium containing the appropriate primary antibodies before fixation. For immunostaining of live, permeabilised MDMs, cells were incubated for 20 min on ice in 0.05% saponin/0.5% BSA/6 µg/ml human IgG/PBS containing the appropriate primary antibodies, washed with ice-cold PBS, fixed, and labelled with secondary antibodies in the presence of 0.1% saponin as described above. Where indicated, live, permeabilised MDMs were immunostained, fixed, and immunostained with additional primary antibodies in the presence of 0.1% Triton X-100 as described above.

### Flow cytometry analysis

For flow cytometry analysis of cell surface Tetherin levels on infected MDMs, cells were incubated for 1 h on ice in complete medium containing 10 µg/ml polyclonal Bst-2 antibody (B02P). Cells were washed once with ice-cold PBS, fixed in 4% PFA, scraped off the tissue culture dish, permeabilised with 0.1% saponin/1% human AB serum/6 µg/ml human IgG/2 mM EDTA/0.05% sodium azide/PBS, labelled for 1 h with α-p24/p55 rabbit serum (ARP432), washed three times in 0.1% saponin/1% human AB serum/2 mM EDTA/0.05% sodium azide/PBS, incubated for 30 min with appropriate Alexa Fluor-conjugated secondary antibodies, washed three times and analysed on an LSR II flow cytometer (BD Biosciences). For flow cytometry analysis of T cells and MDMs following their co-culture, cells were washed with PBS, fixed in 4% PFA, and immunostained with the appropriate primary and subtype specific Alexa Fluor-conjugated secondary antibodies as described above. For flow cytometry analysis of cell surface proteins on primary CD4^+^ T cells, cells were incubated for 30 min at 4°C in 1% FCS/6 µg/ml human IgG/2 mM EDTA/0.05% sodium azide/PBS, labelled for 1 h at 4°C with primary antibodies conjugated to fluorescent dyes, and washed three times in 1% FCS/2 mM EDTA/0.05% sodium azide/PBS. Data were analysed using FlowJo software (TreeStar, Ashland, USA).

To analyse the proportion of dead cells, primary T cells were washed with PBS, labelled with Violet Dead Cell Stain (Life Technologies) according to the manufacturer's instructions, washed with PBS, fixed in 4% PFA, and analysed by flow cytometry.

### Statistical analysis

Unless specified otherwise, p values were calculated using an unpaired Student's t-test.

## Supporting Information

Figure S1 **Tetherin localises to the cell surface, TGN, and IPMCs.** (A,B) Untreated MDMs were incubated for 20 min on ice with 10 µg/ml polyclonal Tetherin (THN) antibody (B02P) and 2.5 µg/ml anti-TGN46, or with 10 µg/ml monoclonal Tetherin antibody (M15) and 2 µg/ml anti-CD9, in the presence of 0.05% saponin. Cells were fixed and labelled with fluorescent secondary antibodies. Arrowheads point at structures reminiscent of IPMCs. Double arrows indicate TGN-like staining patterns. All images are single confocal sections. Scale bars = 10 µm.
(TIF)

Figure S2 **Long-term HIV infection of MDMs triggers an IFN-dependent upregulation of Tetherin.** MDMs were pre-incubated for 30 min at 37°C with 1 µg/ml of IFN-α/β receptor antibody (IFNR AB), or an isotype-matched control antibody (control AB), and subsequently infected with R3A-(+), -(−), or -Udel for seven days in the presence of the same antibodies. All cells were lysed and analysed by western blotting. Numbers above the lanes indicate the Tetherin band intensities relative to uninfected, control antibody-treated MDMs (set at 1).
(TIF)

Figure S3 **Tetherin retains mature HIV on MDMs.** MDMs were infected with R3A-(+), -(−), or -Udel for seven days, fixed, permeabilised, labelled with p24/p55 and p17 Gag antibodies, stained with fluorescent secondary antibodies, and analysed by flow cytometry. Uninfected cell populations were left ungated, infected cell populations gated on the p24/p55 Gag-positive subpopulations, and the p17 Gag fluorescence was analysed. (A) shows the results of a representative experiment, the lines in (B) indicate the average p17 Gag mean fluorescence intensities (MFI) ± SD of duplicate samples from four donors relative to R3A-(+)-infected cells (set at 1). In (B), each donor is represented by differently shaped data points.
(TIF)

Figure S4 **Virological synapses form between HIV-infected MDMs and autologous T cells.** MDMs were infected with HIV-1 BaL for seven days, co-cultured with autologous CD4^+^ T cells for 2.5 h, fixed and immunostained for the indicated proteins. The lower panels show magnifications of the boxed areas in the upper panels. Arrows indicate VS. Scale bar in upper panel = 20 µm, lower panel = 10 µm.
(TIF)

Figure S5 **Tetraspanins and integrins localise to the MDM-T cell VS.** (A–C) MDMs were infected with HIV-1 BaL for seven days, co-cultured with autologous CD4^+^ T cells for 2.5 h, fixed and immunostained for the indicated proteins. Arrows indicate VS. Scale bars = 10 µm.
(TIF)

Figure S6 **HIV-1 R3A spreads more efficiently by cell-cell than by cell-free transmission.** Autologous CD4^+^ T cells were incubated for 6 h with R3A-(+)-infected MDMs, or with cell-free supernatants collected from the same MDMs during the preceding 6 h period. All T cells were collected, and Gag DNA levels in the T cells quantified by qPCR and normalised to GAPDH. For cell-cell transmission, the levels of contaminating MDM-derived Gag and GAPDH DNA were subtracted from the total DNA levels. Bars represent the means ± SD of triplicate samples from a representative experiment relative to cell-free (set at 1).
(TIF)

Figure S7 **Vpu expression in R3A-infected MDMs does not influence their adhesion to T cells.** (A) R3A-(+), -(−), or -Udel-infected MDMs, or uninfected control MDMs, were co-cultured with autologous CD4^+^ T cells for 6 h. T cells were then washed off the MDMs with PBS, fixed and counted. Bars represent the mean proportions of recovered T cells ± SD of duplicate samples from three donors. (B–E) R3A-infected MDMs, or uninfected control MDMs, were co-cultured with autologous CD4^+^ T cells for 6 h. No T cells were added to uninfected MDMs as a control. T cells were washed off the MDMs with PBS. The MDMs were fixed with PFA, immunostained for the T cell marker CD3, the MDM marker CD68, and HIV-1 p24/p55 Gag in the presence of 0.1% saponin, and analysed by flow cytometry. (B) shows CD3/CD68 plots from a representative experiment, and the numbers within the gates indicate the relative frequencies of MDMs (set at 1), and T cells that had detached during the staining procedure and had therefore loosely interacted with MDMs. The bars in (C) represent the mean ratios of T cells to MDMs ± SD of duplicate samples from four donors. (D) shows CD3 plots of CD68-positive, uninfected or infected MDMs from a representative experiment, and the gates are set to discriminate between MDMs without T cells and MDMs that had remained associated with T cells during the staining procedure, and had therefore formed tight interactions. (E) shows the mean proportions of MDMs that had formed tight interactions with T cells ± SD of duplicate samples from three donors, and each donor is represented by differently shaped data points.
(TIF)

Figure S8 **R3A-infected MDMs may form transient VS.** (A) MDMs were infected with HIV-1 R3A for seven days, co-cultured with autologous CD4^+^ T cells for 2.5 h, fixed and immunostained for the indicated proteins. Scale bar = 10 µm. (B–C) Unpermeabilised primary CD4^+^ T cells were immunostained for the indicated proteins and analysed by flow cytometry. (B) shows the results from a representative experiment. The red graphs represent stained T cells, the grey graphs unstained control cells. (C) shows the mean proportions of positive cells ± SD from four donors, where each donor is represented by differently shaped data points.
(TIF)

Figure S9 **Western blotting conditions effect Tetherin quantification.** MDMs were stimulated for 24 h with 0–2,721 U/ml (0–10 ng/ml) IFN-β eight days post isolation from buffy coats, and lysed in non-reducing Laemmli buffer. Untreated lysates (non-reduced) and lysates treated with 2-mercaptoethanol (reduced) were separated on SDS-polyacrylamide gels, and Tetherin levels were analysed by western blotting.
(TIF)

Figure S10 **T cells remain viable after their co-culture with HIV-infected MDMs.** (A) To confirm the reactivity of the dead cell stain, primary CD4^+^ T cells from one donor were incubated for 20 min at 60°C, labelled with dead cell stain, fixed, and analysed by flow cytometry. Cells were kept at 37°C and labelled with the same stain (37°C), or left unlabelled (no stain), as controls. (B–C) R3A-(+), -(−), or -Udel-infected MDMs, or uninfected control MDMs, were co-cultured with autologous CD4^+^ T cells for 6 h. T cells were washed off the MDMs with PBS, and labelled with a Violet Dead Cell Stain immediately, or after another 6-, 18-, or 30 h-incubation. The cells were fixed, immunostained for CD3, and analysed by flow cytometry. (B) shows the results of a representative experiment, and the numbers in the top right quadrants indicate the proportions of dead CD3^+^ cells. (C) shows the mean proportions of dead CD3^+^ cells ± SD from duplicate samples of three donors.
(TIF)

Figure S11 **MDMs infected with R3A-(+), -(−) and –Udel for seven days show similar infection levels.** MDMs were infected with R3A-(+), -(−), or -Udel for seven days. (A–B) The cells were lysed, and analysed by western blotting. The blots in (A) and (B) are from parallel cultures of the MDMs used for the cell-cell transmission experiments shown in Fig. 9B and Fig. 9C, respectively. (C–D) The cells were fixed, permeabilised, labelled with p24/p55 Gag antibodies, and stained with fluorescent secondary antibodies. The proportions of p24/p55 Gag-positive MDMs were analysed by flow cytometry. (A) shows the results of a representative experiment, the lines in (B) indicate the means of duplicate samples from four donors, where each donor is represented by differently shaped data points. Note that the analyses shown in (C) and (D) were performed on the same samples used for Fig. S3.
(TIF)
