# Supplementary figures and images for: Tetherin Can Restrict Cell-Free and Cell-Cell Transmission of HIV from Primary Macrophages to T Cells
Source: PLoS Pathog. 2014 Jul 3;10(7):e1004189. doi: 10.1371/journal.ppat.1004189 (PMC4081785; doi:10.1371/journal.ppat.1004189)

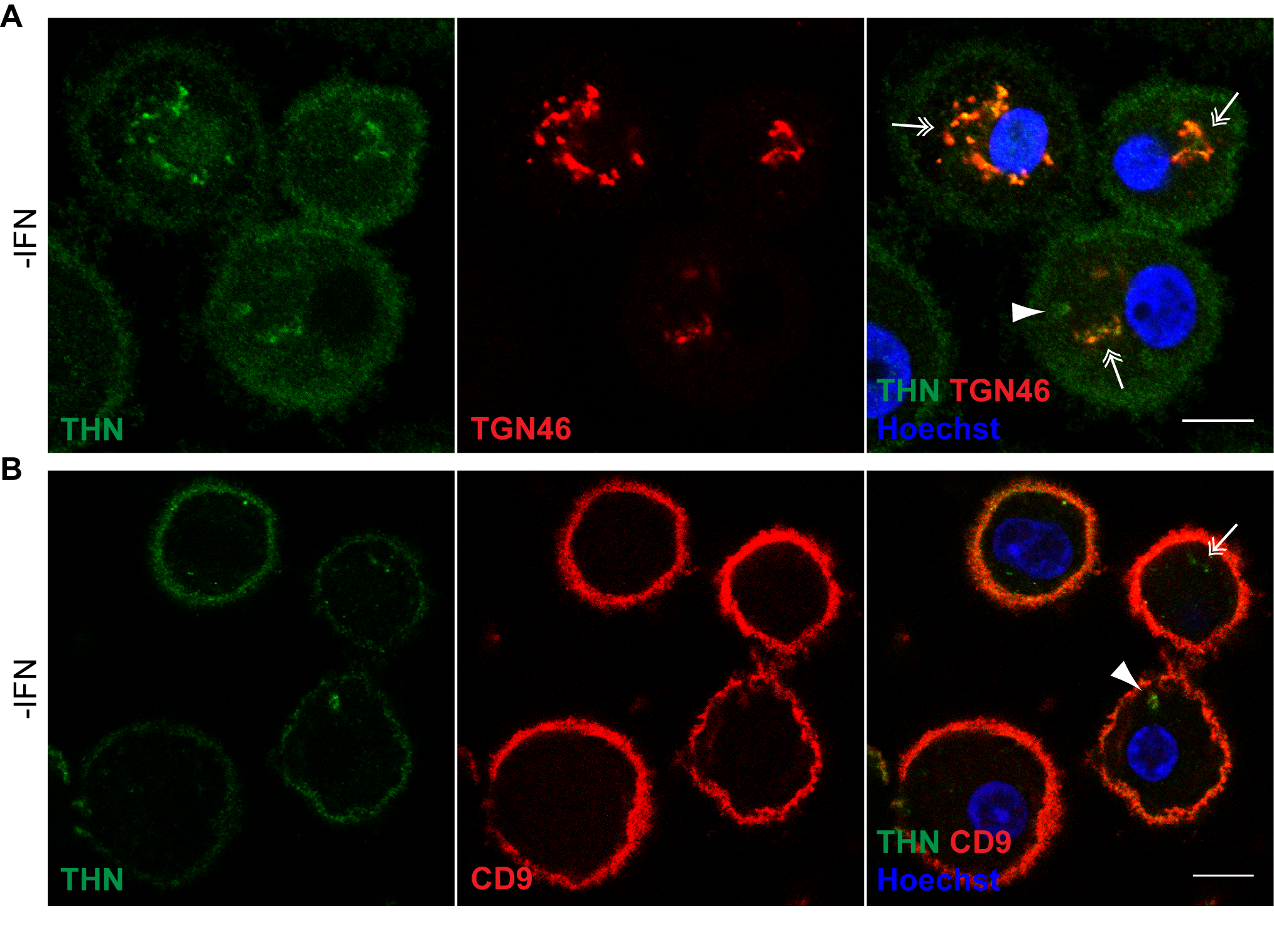

Supplement: Figure S1 — Tetherin localises to the cell surface, TGN, and IPMCs. (A,B) Untreated MDMs were incubated for 20 min on ice with 10 µg/ml polyclonal Tetherin (THN) antibody (B02P) and 2.5 µg/ml anti-TGN46, or with 10 µg/ml monoclonal Tetherin antibody (M15) and 2 µg/ml anti-CD9, in the presence of 0.05% saponin. Cells were fixed and labelled with fluorescent secondary antibodies. Arrowheads point at structures reminiscent of IPMCs. Double arrows indicate TGN-like staining patterns. All images are single confocal sections. Scale bars = 10 µm. (TIF) [file ppat.1004189.s001.tif]

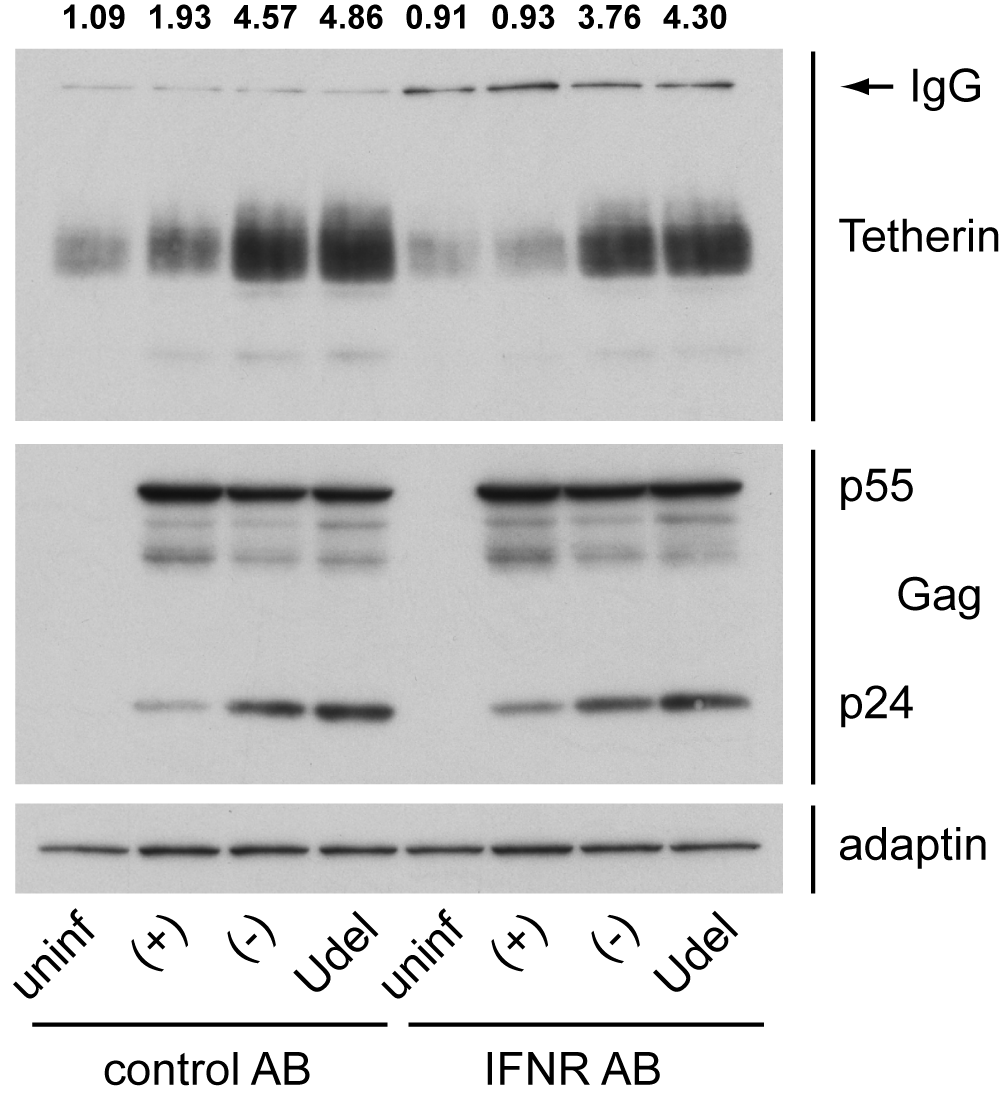

Supplement: Figure S2 — Long-term HIV infection of MDMs triggers an IFN-dependent upregulation of Tetherin. MDMs were pre-incubated for 30 min at 37°C with 1 µg/ml of IFN-α/β receptor antibody (IFNR AB), or an isotype-matched control antibody (control AB), and subsequently infected with R3A-(+), -(−), or -Udel for seven days in the presence of the same antibodies. All cells were lysed and analysed by western blotting. Numbers above the lanes indicate the Tetherin band intensities relative to uninfected, control antibody-treated MDMs (set at 1). (TIF) [file ppat.1004189.s002.tif]

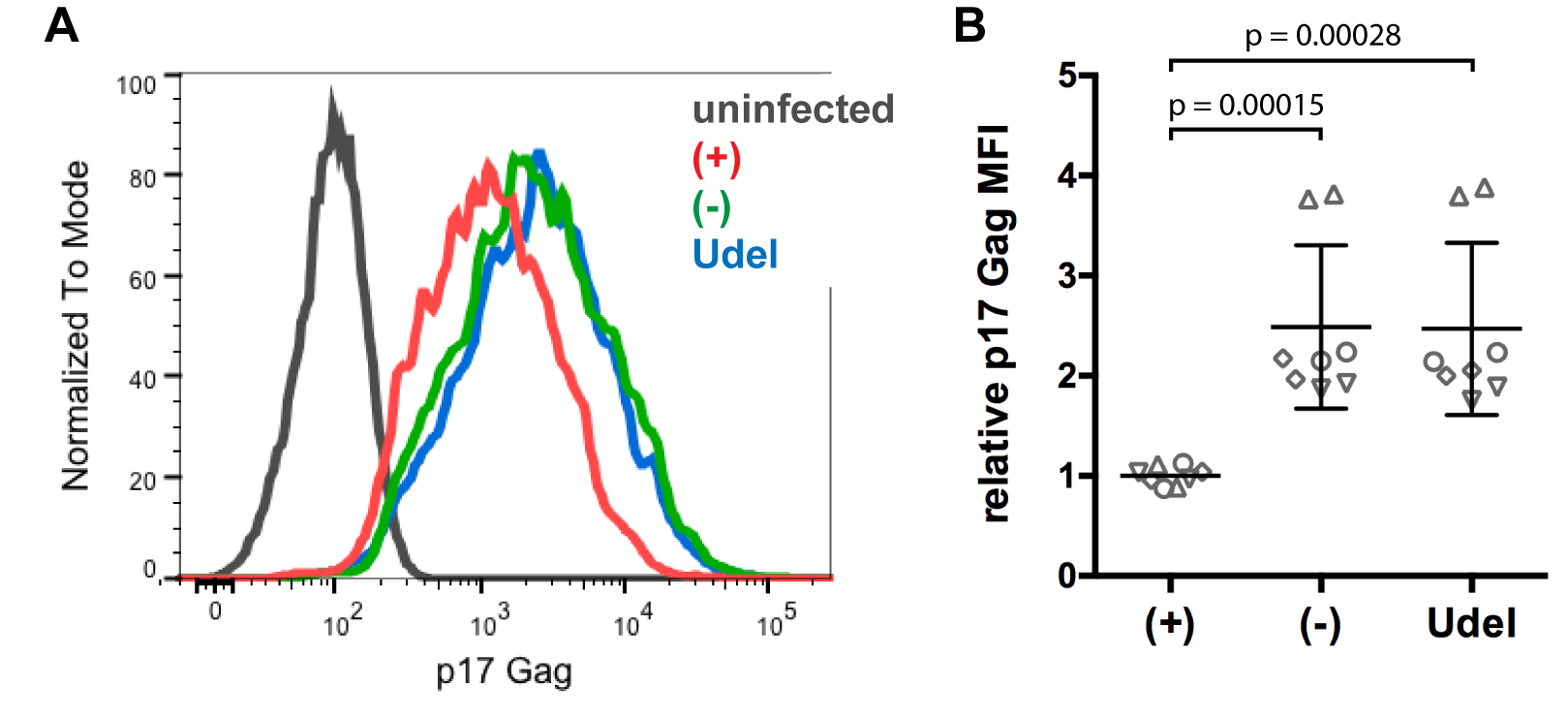

Supplement: Figure S3 — Tetherin retains mature HIV on MDMs. MDMs were infected with R3A-(+), -(−), or -Udel for seven days, fixed, permeabilised, labelled with p24/p55 and p17 Gag antibodies, stained with fluorescent secondary antibodies, and analysed by flow cytometry. Uninfected cell populations were left ungated, infected cell populations gated on the p24/p55 Gag-positive subpopulations, and the p17 Gag fluorescence was analysed. (A) shows the results of a representative experiment, the lines in (B) indicate the average p17 Gag mean fluorescence intensities (MFI) ± SD of duplicate samples from four donors relative to R3A-(+)-infected cells (set at 1). In (B), each donor is represented by differently shaped data points. (TIF) [file ppat.1004189.s003.tif]

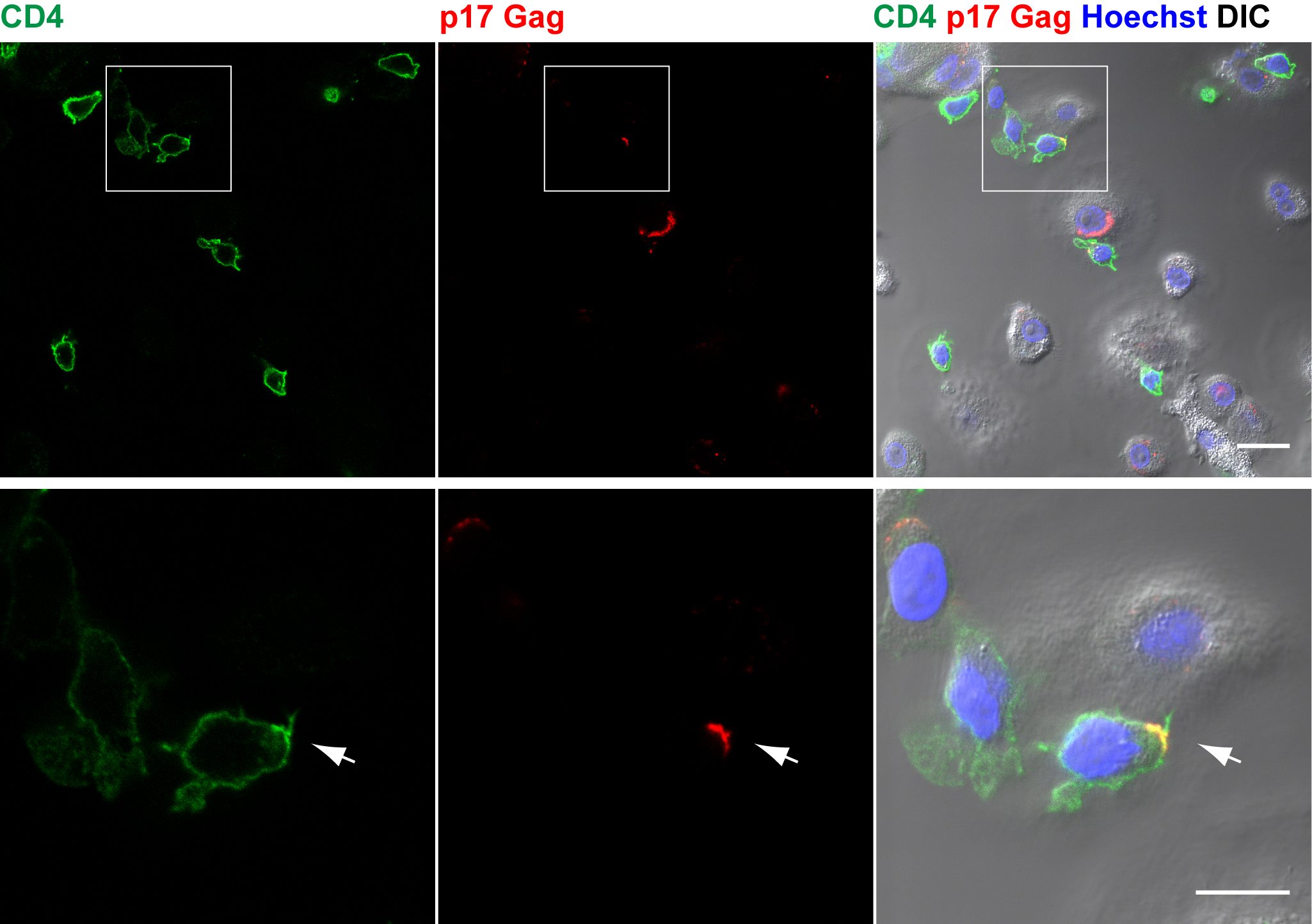

Supplement: Figure S4 — Virological synapses form between HIV-infected MDMs and autologous T cells. MDMs were infected with HIV-1 BaL for seven days, co-cultured with autologous CD4+ T cells for 2.5 h, fixed and immunostained for the indicated proteins. The lower panels show magnifications of the boxed areas in the upper panels. Arrows indicate VS. Scale bar in upper panel = 20 µm, lower panel = 10 µm. (TIF) [file ppat.1004189.s004.tif]

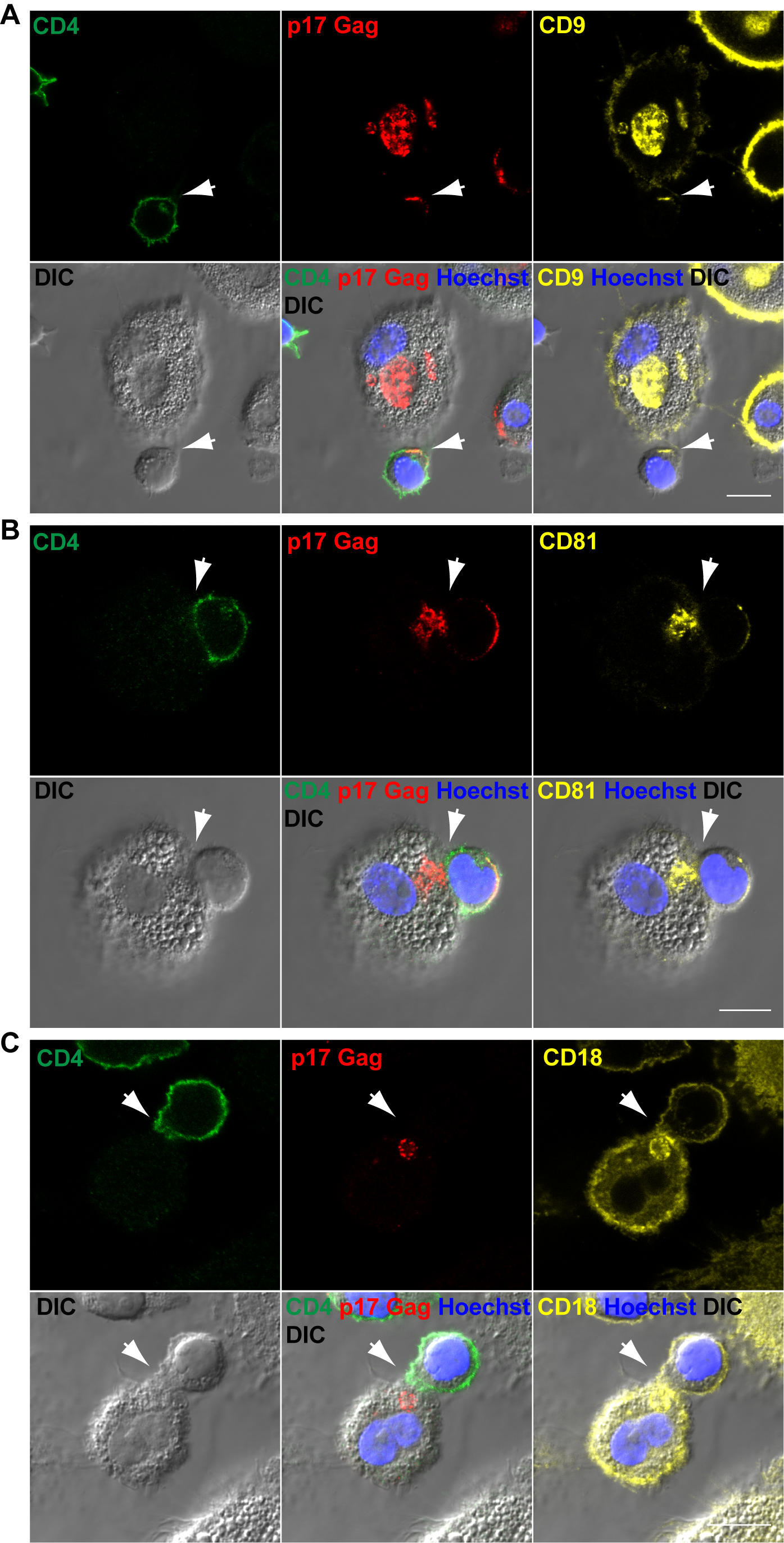

Supplement: Figure S5 — Tetraspanins and integrins localise to the MDM-T cell VS. (A–C) MDMs were infected with HIV-1 BaL for seven days, co-cultured with autologous CD4+ T cells for 2.5 h, fixed and immunostained for the indicated proteins. Arrows indicate VS. Scale bars = 10 µm. (TIF) [file ppat.1004189.s005.tif]

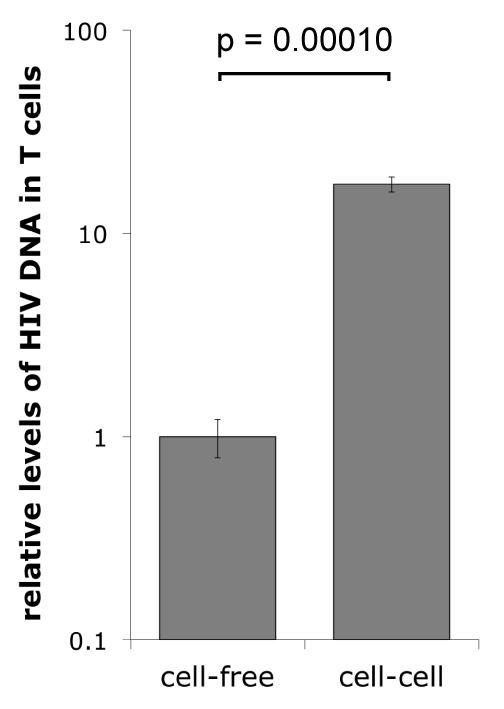

Supplement: Figure S6 — HIV-1 R3A spreads more efficiently by cell-cell than by cell-free transmission. Autologous CD4+ T cells were incubated for 6 h with R3A-(+)-infected MDMs, or with cell-free supernatants collected from the same MDMs during the preceding 6 h period. All T cells were collected, and Gag DNA levels in the T cells quantified by qPCR and normalised to GAPDH. For cell-cell transmission, the levels of contaminating MDM-derived Gag and GAPDH DNA were subtracted from the total DNA levels. Bars represent the means ± SD of triplicate samples from a representative experiment relative to cell-free (set at 1). (TIF) [file ppat.1004189.s006.tif]

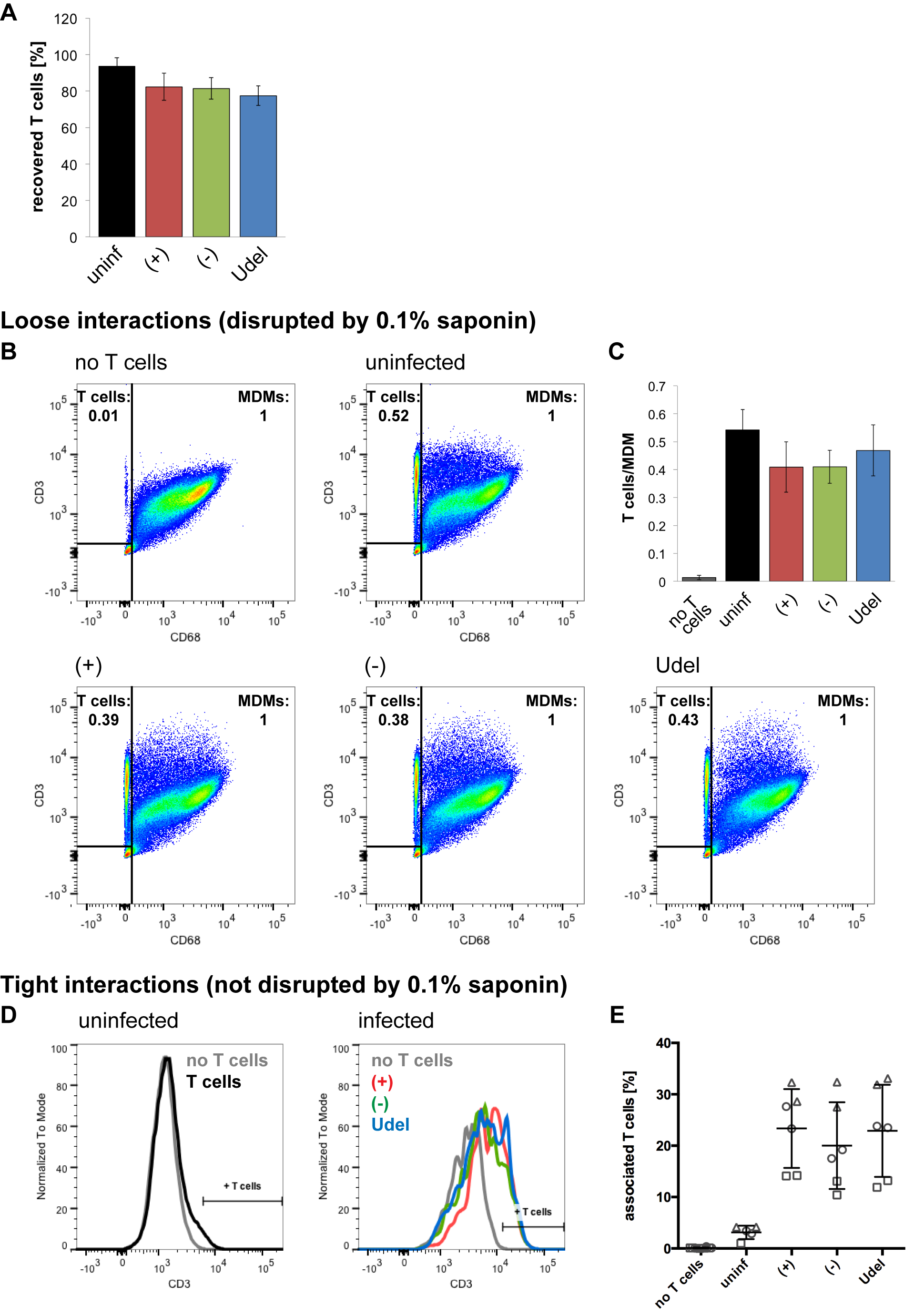

Supplement: Figure S7 — Vpu expression in R3A-infected MDMs does not influence their adhesion to T cells. (A) R3A-(+), -(−), or -Udel-infected MDMs, or uninfected control MDMs, were co-cultured with autologous CD4+ T cells for 6 h. T cells were then washed off the MDMs with PBS, fixed and counted. Bars represent the mean proportions of recovered T cells ± SD of duplicate samples from three donors. (B–E) R3A-infected MDMs, or uninfected control MDMs, were co-cultured with autologous CD4+ T cells for 6 h. No T cells were added to uninfected MDMs as a control. T cells were washed off the MDMs with PBS. The MDMs were fixed with PFA, immunostained for the T cell marker CD3, the MDM marker CD68, and HIV-1 p24/p55 Gag in the presence of 0.1% saponin, and analysed by flow cytometry. (B) shows CD3/CD68 plots from a representative experiment, and the numbers within the gates indicate the relative frequencies of MDMs (set at 1), and T cells that had detached during the staining procedure and had therefore loosely interacted with MDMs. The bars in (C) represent the mean ratios of T cells to MDMs ± SD of duplicate samples from four donors. (D) shows CD3 plots of CD68-positive, uninfected or infected MDMs from a representative experiment, and the gates are set to discriminate between MDMs without T cells and MDMs that had remained associated with T cells during the staining procedure, and had therefore formed tight interactions. (E) shows the mean proportions of MDMs that had formed tight interactions with T cells ± SD of duplicate samples from three donors, and each donor is represented by differently shaped data points. (TIF) [file ppat.1004189.s007.tif]

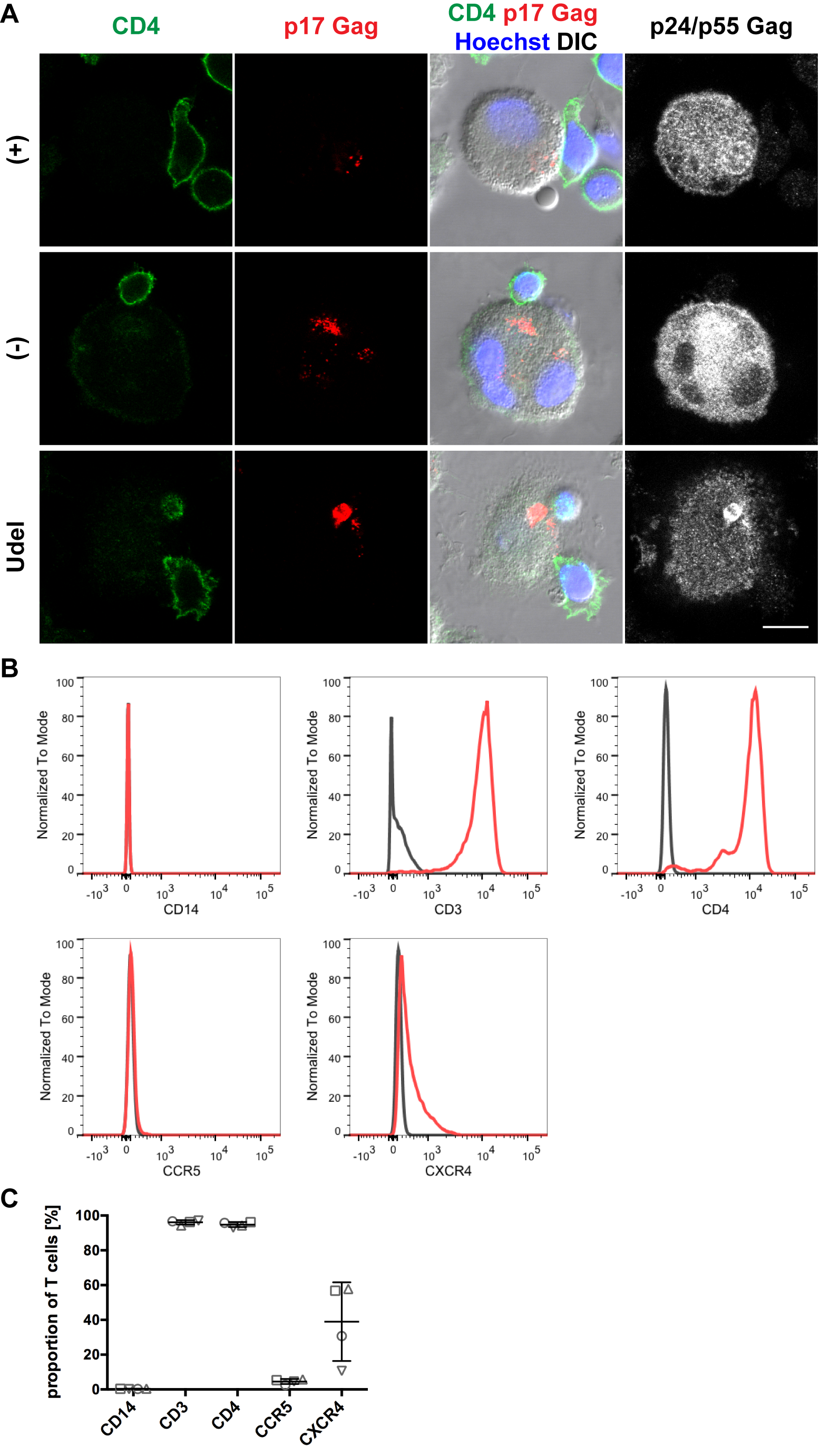

Supplement: Figure S8 — R3A-infected MDMs may form transient VS. (A) MDMs were infected with HIV-1 R3A for seven days, co-cultured with autologous CD4+ T cells for 2.5 h, fixed and immunostained for the indicated proteins. Scale bar = 10 µm. (B–C) Unpermeabilised primary CD4+ T cells were immunostained for the indicated proteins and analysed by flow cytometry. (B) shows the results from a representative experiment. The red graphs represent stained T cells, the grey graphs unstained control cells. (C) shows the mean proportions of positive cells ± SD from four donors, where each donor is represented by differently shaped data points. (TIF) [file ppat.1004189.s008.tif]

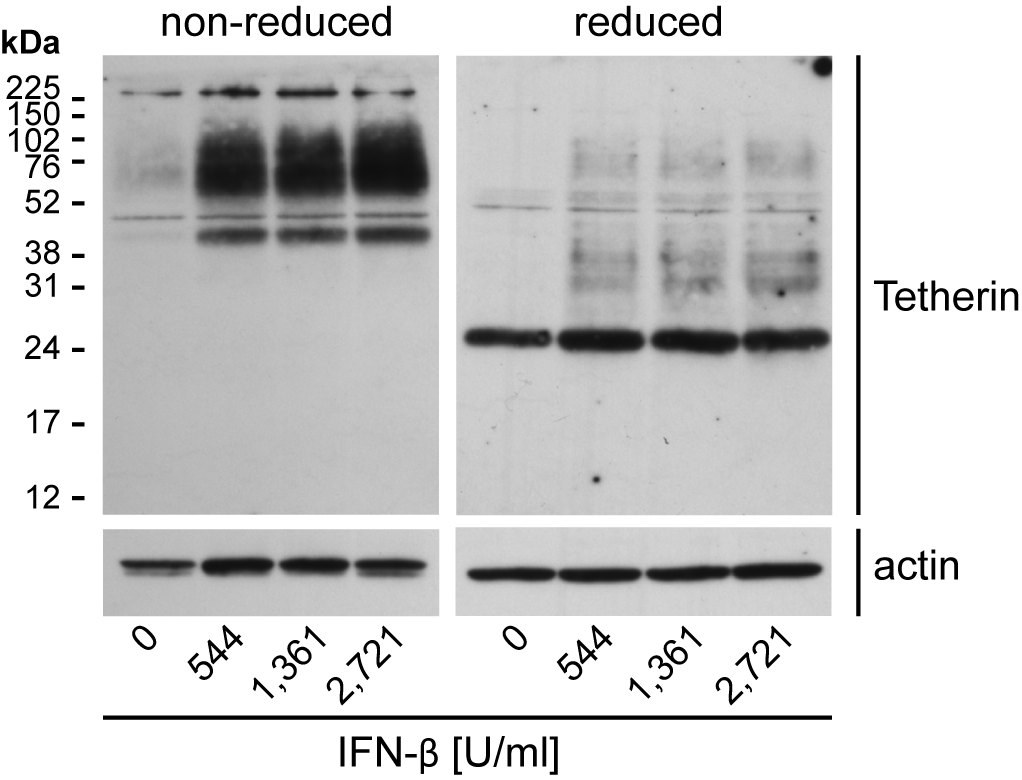

Supplement: Figure S9 — Western blotting conditions effect Tetherin quantification. MDMs were stimulated for 24 h with 0–2,721 U/ml (0–10 ng/ml) IFN-β eight days post isolation from buffy coats, and lysed in non-reducing Laemmli buffer. Untreated lysates (non-reduced) and lysates treated with 2-mercaptoethanol (reduced) were separated on SDS-polyacrylamide gels, and Tetherin levels were analysed by western blotting. (TIF) [file ppat.1004189.s009.tif]

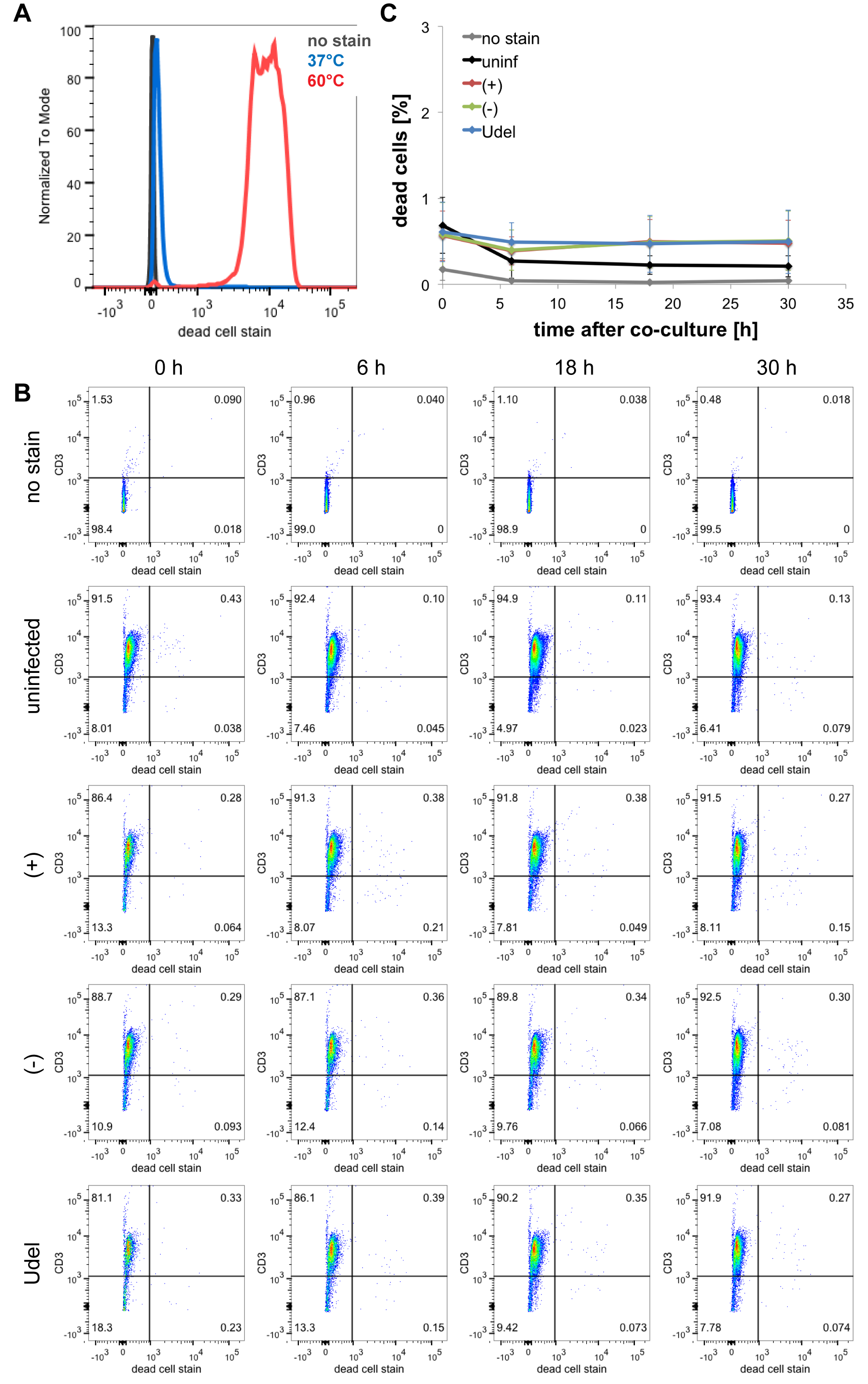

Supplement: Figure S10 — T cells remain viable after their co-culture with HIV-infected MDMs. (A) To confirm the reactivity of the dead cell stain, primary CD4+ T cells from one donor were incubated for 20 min at 60°C, labelled with dead cell stain, fixed, and analysed by flow cytometry. Cells were kept at 37°C and labelled with the same stain (37°C), or left unlabelled (no stain), as controls. (B–C) R3A-(+), -(−), or -Udel-infected MDMs, or uninfected control MDMs, were co-cultured with autologous CD4+ T cells for 6 h. T cells were washed off the MDMs with PBS, and labelled with a Violet Dead Cell Stain immediately, or after another 6-, 18-, or 30 h-incubation. The cells were fixed, immunostained for CD3, and analysed by flow cytometry. (B) shows the results of a representative experiment, and the numbers in the top right quadrants indicate the proportions of dead CD3+ cells. (C) shows the mean proportions of dead CD3+ cells ± SD from duplicate samples of three donors. (TIF) [file ppat.1004189.s010.tif]

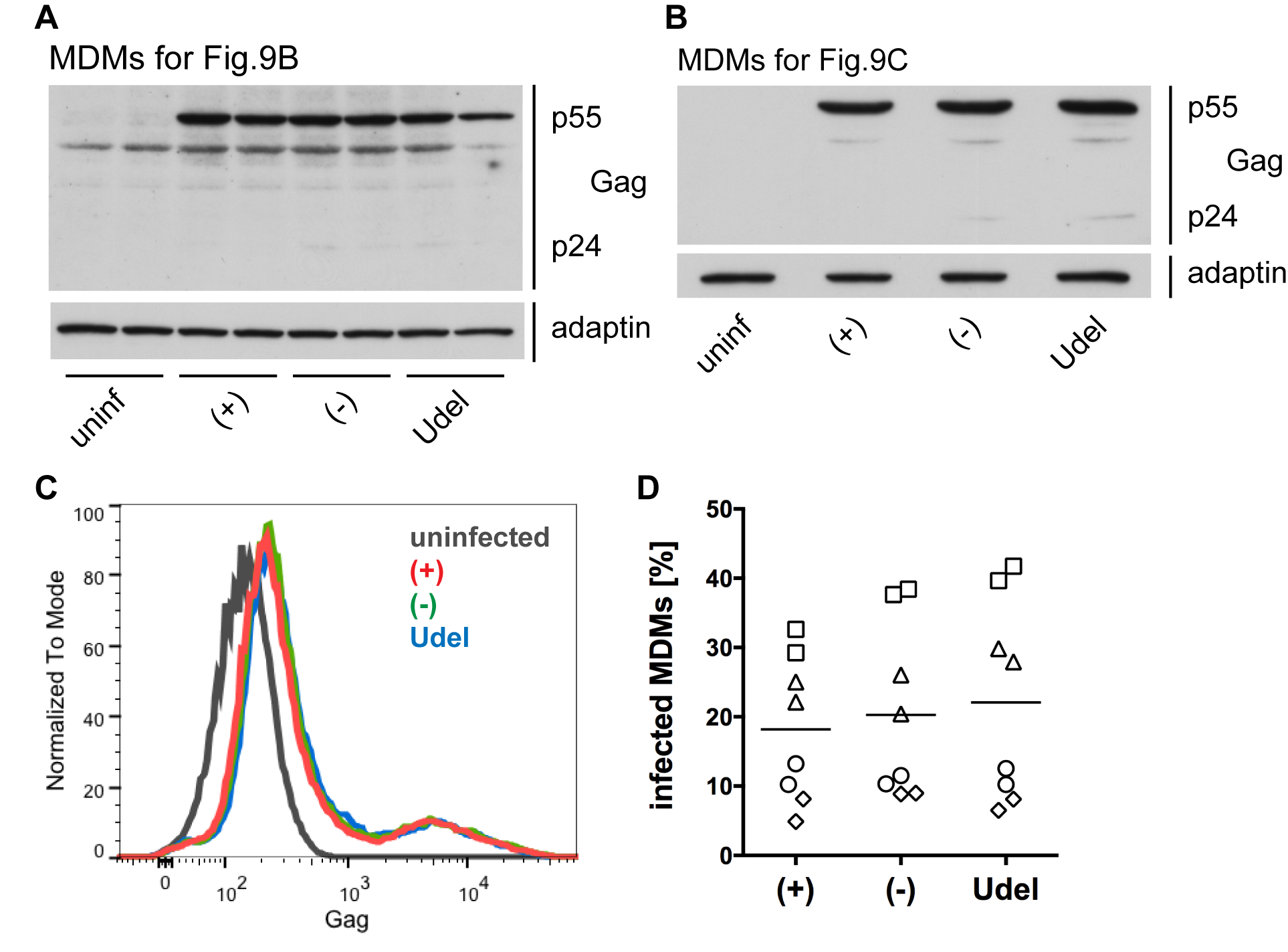

Supplement: Figure S11 — MDMs infected with R3A-(+), -(−) and –Udel for seven days show similar infection levels. MDMs were infected with R3A-(+), -(−), or -Udel for seven days. (A–B) The cells were lysed, and analysed by western blotting. The blots in (A) and (B) are from parallel cultures of the MDMs used for the cell-cell transmission experiments shown in Fig. 9B and Fig. 9C, respectively. (C–D) The cells were fixed, permeabilised, labelled with p24/p55 Gag antibodies, and stained with fluorescent secondary antibodies. The proportions of p24/p55 Gag-positive MDMs were analysed by flow cytometry. (A) shows the results of a representative experiment, the lines in (B) indicate the means of duplicate samples from four donors, where each donor is represented by differently shaped data points. Note that the analyses shown in (C) and (D) were performed on the same samples used for Fig. S3. (TIF) [file ppat.1004189.s011.tif]
